# Rattan‐Based Solar Evaporator Hits 3.34 Kg·M^−2^·h^−1^ and Long‐Term Salt Resistance via Programmed Carbonization

**DOI:** 10.1002/advs.202511782

**Published:** 2025-10-24

**Authors:** Shanshan Jia, Xiaqing Qin, Shijie Dai, Yan Qing, Jianwu Dai, Shaobo Zhang, Hui Xiao, Yuzhu Chen, Jinqiu Qi, Yongqian Yang, Yao Lu, Zhiping Su, Yong Zhuo, De Wu

**Affiliations:** ^1^ College of Forestry Sichuan Agricultural University Chengdu 611130 P. R. China; ^2^ Forest Ecology and Conservation in the Upper Reaches of the Yangtze River Key Laboratory of Sichuan Province Chengdu 610000 P. R. China; ^3^ College of Materials Science and Engineering Central South University of Forestry and Technology Changsha 410004 P. R. China; ^4^ College of Mechanical and Electrical Engineering Sichuan Agricultural University Ya'an 625014 P. R. China; ^5^ Animal Nutrition Institute Sichuan Agricultural University Chengdu 611130 P. R. China

**Keywords:** biomass, high stability, programmed carbonization, rattan, solar evaporator, versatile application

## Abstract

Global water scarcity urgently demands sustainable purification solutions. A high‐performance solar evaporator crafted entirely from renewable biomass is reported, e.g., rattan, through an innovative programmed carbonization process. By applying controlled gradient heating, bimodal hierarchical porous structures are engineered within the plant material that optimize water transport, heat localization, and vaporization energy reduction. This yields a record evaporation rate of 3.34 kg m^−2^ h^−1^ under 1 sun, which is 1.69× faster than unprogrammed carbonization. Critically, the design achieves exceptional durability, withstanding continuous outdoor evaporation for over 14 days in high‐salinity brine (20 wt.% NaCl) without noticeable salt accumulation or microbial growth. Outdoor experiments further confirm its stable daily fresh water production (≈24.49 L) with high quality that meets World Health Organization (WHO) drinking standards. This work offers a scalable, eco‐friendly path to freshwater access, particularly for off‐grid communities, by leveraging globally abundant plants and requiring only sunlight.

## Graphical abstract

A rattan‐based evaporator with bimodal hierarchical porous structures achieves an high evaporation rate of 3.34 kg m^−2^ h^−1^ under 1 sun illumination by optimizing the surface water layer, localizing heat, and reducing vaporization enthalpy.

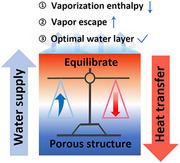

## 1 Introduction

Freshwater scarcity has escalated into a global crisis, threatening sustainable development through the compounding pressures of population growth, climate disruption, and industrial expansion.^[^ ^1^, ^2^, ^3^, ^4^ ^]^ Recent assessments reveal that 2.2 billion people lack access to safely managed drinking water according to United Nations Water (UN‐Water), with a deficit projected to intensify as climate‐driven droughts expand, with 5 billion individuals anticipated to face water shortages by 2050.^[^ ^1^, ^2^ ^]^ The socioeconomic toll is equally alarming: unchecked scarcity could erode 8% of global GDP by mid‐century, disproportionately affecting vulnerable regions where losses may exceed 15%.^[^ ^3^ ^]^ Against this urgent backdrop, interfacial solar evaporation emerges as a promising decentralized solution, leveraging sunlight to desalinate seawater and purify contaminated sources.^[^ ^4^, ^5^, ^6^, ^7^, ^8^ ^]^ However, the widespread deployment of this technology remains constrained by fundamental limitations in existing solar evaporators.

Conventional biomass‐based solar evaporators (BBSEs), while leveraging natural porosity and hydrophilicity for water transport,^[^ ^9^, ^10^, ^11^, ^12^, ^13^, ^14^ ^]^ typically achieve evaporation rates around 1.5 kg m^−2^ h^−1^ under 1 sun illumination;^[^ ^15^, ^16^ ^]^ they are usually insufficient for practical water production. Existing strategies for enhancing evaporation efficiency are typically achieved through engineering structures, integration of functional materials, and application of external heat to optimize thermal management, regulating optical absorption, and lowering the enthalpy of water evaporation.^[^ ^17^, ^18^, ^19^, ^20^, ^21^ ^]^ The integration of functional materials such as metal‐organic frameworks or carbon quantum dots has been shown to increase evaporation rates above 3.0 kg m^−2^ h^−1^.^[^ ^22^, ^23^ ^]^ Similarly, coupling with external heat input like Joule heating can further improve performance.^[^ ^24^ ^]^ However, these approaches often suffer from complex fabrication, high cost, interfacial mismatch, and limited long‐term stability. Structure‐engineering‐based strategies, such as porous architectures optimization and vertical aligned channels, typically achieve enhancement without the need for functional materials or external heat input.^[^ ^25^, ^26^ ^]^ However, most as‐prepared biomass‐based evaporators exhibit evaporation rates approximately 2 kg m^−2^ h^−1^ under 1 sun,^[^ ^27^, ^28^, ^29^, ^30^ ^]^ which remains significantly lower than the rates exceeding 4 kg m^−2^ h^−1^ in other evaporator systems, such as hydrogel‐based evaporator.^[^ ^31^, ^32^ ^]^

One primary reason is that water desalination is highly energy‐intensive. Since diffuse sunlight under natural conditions is limited, it barely meets the energy required for evaporation. Hydrogel‐based evaporators achieve high evaporation rates by reducing the vaporization enthalpy of water through tailored polymer‐water interactions,^[^ ^33^ ^]^ thereby circumventing the limitations of solar energy input. Moreover, natural biomass is inherently hydrophilic and porous, which facilitates high water flux but may hinder evaporation due to excessive water accumulation on the surface, leading to increased heat loss.^[^ ^34^ ^]^ The enthalpy of vaporization, water flux, and heat localization highly depend on porous structure, however, the precise regulation of porous architecture and its relationships with evaporation performance remain largely unaddressed, particularly in biomass‐based evaporators derived from bulk materials.

Their operational lifespan is further curtailed by salt accumulation and microbial fouling.^[^ ^35^, ^36^, ^37^, ^38^ ^]^ While strategies such as Janus structuring, hydrophobic modification, liquid convection, and localized crystallization can alleviate salt accumulation, they often fail to sustain high evaporation rates.^[^ ^15^ ^]^ Similarly, mildew resistance approaches based on antimicrobial nanomaterials (e.g., silver, zinc oxide, nanoparticles, and MXene) increase fabrication cost and complexity, and raise environmental concerns due to potential leaching.^[^ ^27^ ^]^ For example, Ren et al. employed carbon fiber powder and aramid fiber powder to engineer Janus structure, enabling solar evaporators with salt‐resistant properties and evaporation rate of 1.25 kg m^−2^ h^−1^ at a salinity of 15 wt.% brine under 1 sun.^[^ ^39^ ^]^ Xu et al. used graphene flake/polyaniline nanocomposite to fabricate hydrophobic surface on wood sponge, achieving salt‐resistant property and evaporation rate of 1.49 kg m^−2^ h^−1^ under 1 sun.^[^ ^40^ ^]^ Song et al. synthesized Ag nanoparticles using lignin as reductant, subsequently incorporated them into a hydrogel, achieving an evaporation rate of 1.85 kg m^−2^ h^−1^ under 1 sun irradiation while maintaining stable performance for > 30 days.^[^ ^41^ ^]^ Existing studies have demonstrated that structural engineering of bulk biomass can enhance salt resistance, mildew inhibition, and evaporation rates without the need for functional additives or external energy.^[^ ^25^, ^26^, ^42^ ^]^ However, integrating all these properties simultaneously remains challenging. These challenges highlight the need for BBSEs combining efficiency, durability, and low cost.

In this work, we developed a programmed carbonization strategy that transforms globally abundant biomass, such as rattan, balsa wood, and crop residues, into high‐performance evaporators. By engineering bimodal hierarchical pore structures via gradient thermal treatment, we optimize the competing demands of water flux, heat localization, and vaporization enthalpy reduction, enabling an unprecedented evaporation rate of 3.34 kg m^−2^ h^−1^ under 1 sun illumination. Self‐regenerating channels prevent salt accumulation, while the removal of starch and lignocellulosic nutrients imparts mildew resistance. These combined features of high flux, salt stability, and microbial resistance enable over 14 days of continuous outdoor evaporation in 20 wt.% NaCl brine without noticeable salt crystallization or microbial contamination.

## 2 Results and Discussion

**Figure 1:**
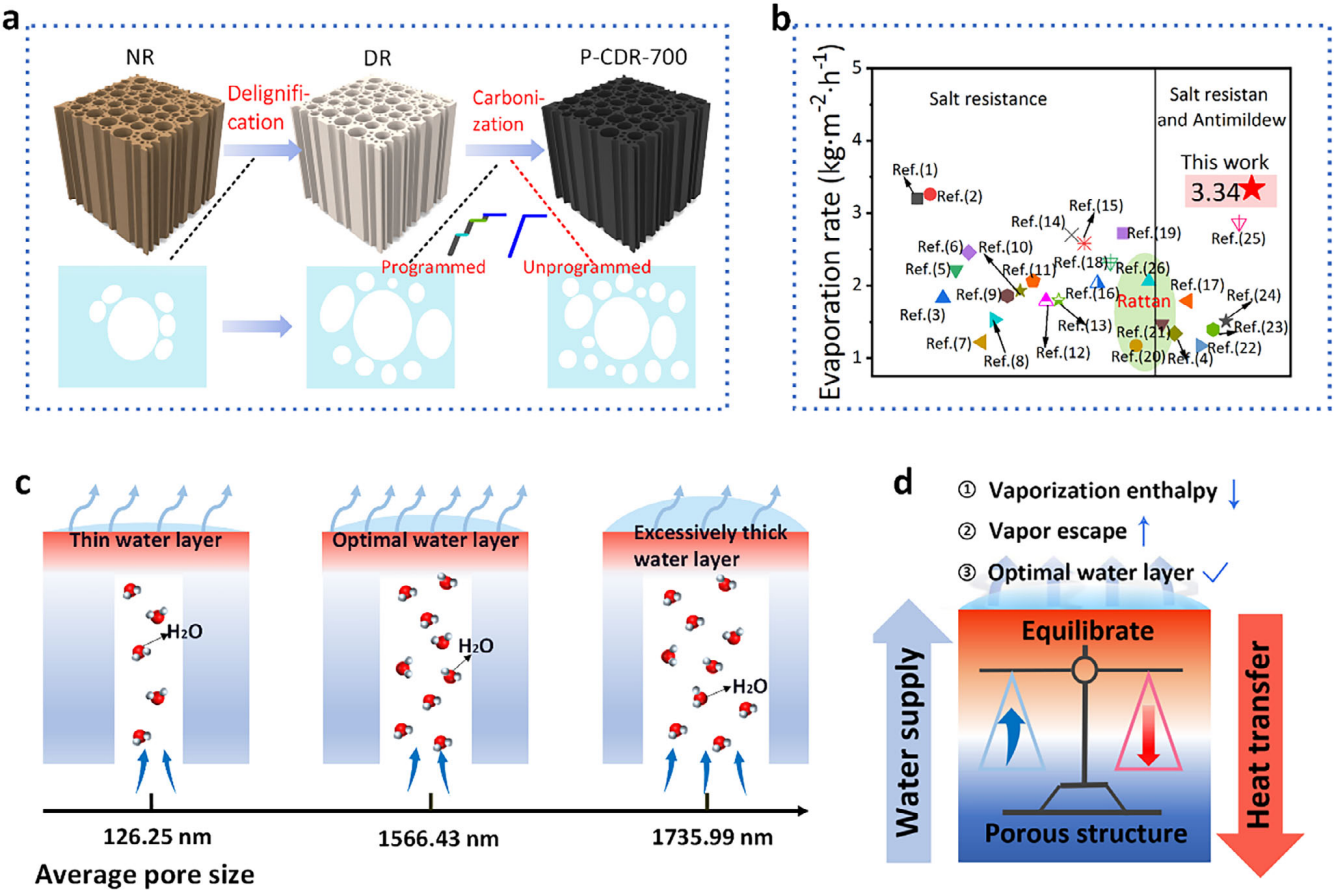
a) The process for the selective delignification and the relationship between pore structures and carbonization methods. b) The advantage of our evaporator compared with other biomass‐based evaporators (For detailed references, see Table S1, Supporting Information). c) The effect of porous structure on water content, water layer thickness, and evaporation rate. d) Programmed optimal structure achieving reduced vaporization enthalpy, lower vapor escape resistance, and optimal water layer (i.e., water/heat transfer balance).

The P‐CDR‐700 was fabricated through soft delignification followed by a programmed carbonization process under a nitrogen atmosphere (**Figure** **1**a; Figure S1, Supporting Information). Programmed carbonization contributes to an optimal balance between water transport and heat localization, rapid vapor escape, and decreased evaporation enthalpy, and thus results in a high evaporation rate (Figure 1c,d). Additionally, programmed carbonization processing also significantly enhances mildew resistance and mechanical robustness, which are crucial for ensuring long‐term durability. Soft delignification could also improve evaporation performance and durability; however, its contribution is less significant than that of programmed carbonization in this system, this conclusion is quantitatively verified in following section. The evaporation rate of our BBSE surpasses that of most biomass‐based evaporators, including the majority of carbonized plant‐derived devices, and represents the highest value reported to date for rattan‐based systems (Figure 1b). More importantly, the same performance can be consistently achieved across different batches of samples (Figures S2 and S3, Supporting Information).

**Figure 2:**
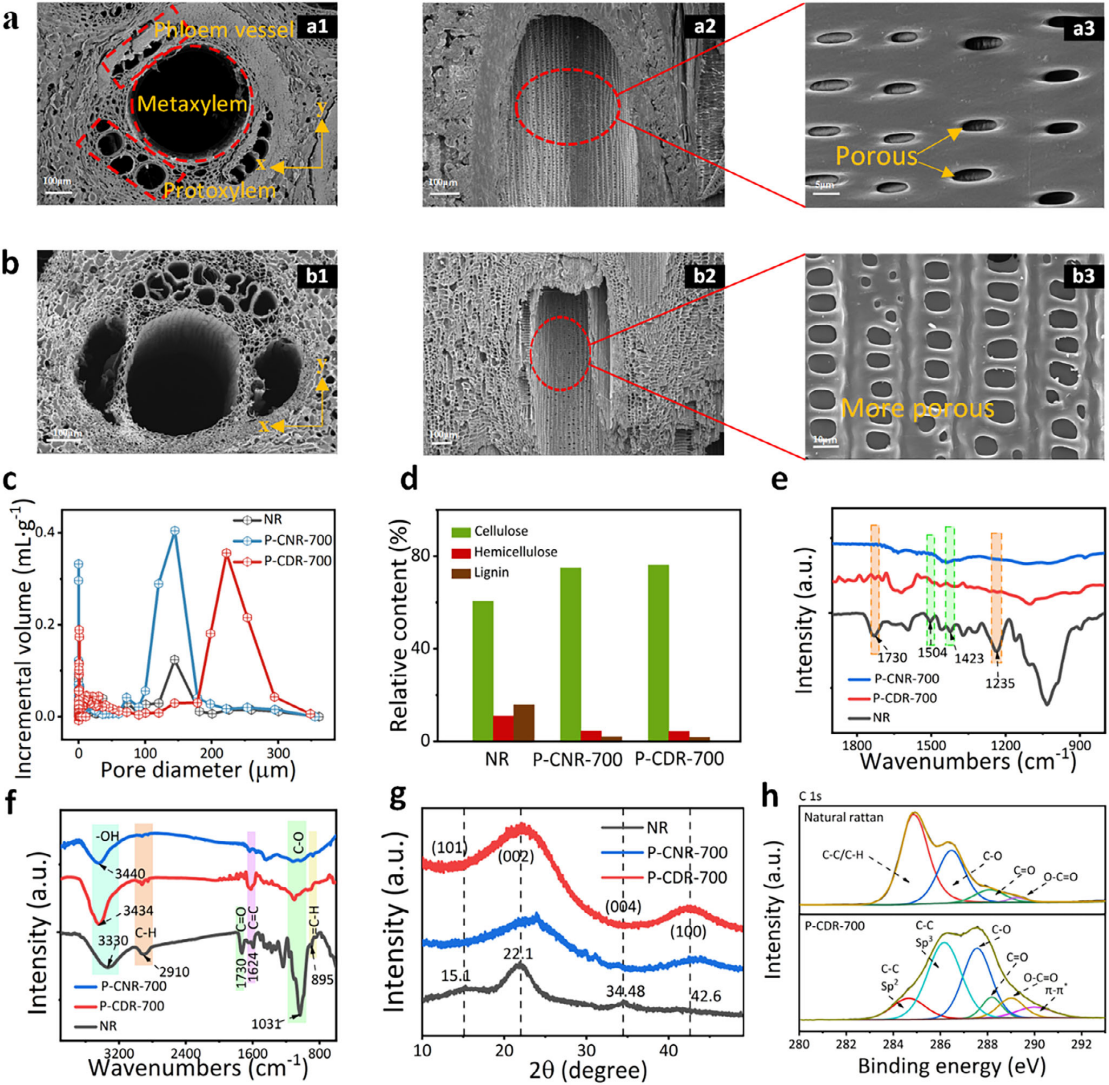
a) The SEM images of NR (a1 for the transverse section, a2 and a3 for the longitudinal section. b) The SEM images of P‐CDR‐700 (b1 for the transverse section, b2 and b3 for the longitudinal section. c) The pore size distribution of NR, P‐CNR‐700, and P‐CDR‐700. d) The relative content of cellulose, hemicellulose, and lignin in NR and P‐CDR‐700. e,f) The FT‐IR spectra and g) the XRD spectra of NR, P‐CNR‐700, and P‐CDR‐700. h) The XPS fine spectra of C 1s of NR and P‐CDR‐700.

**Figure 3:**
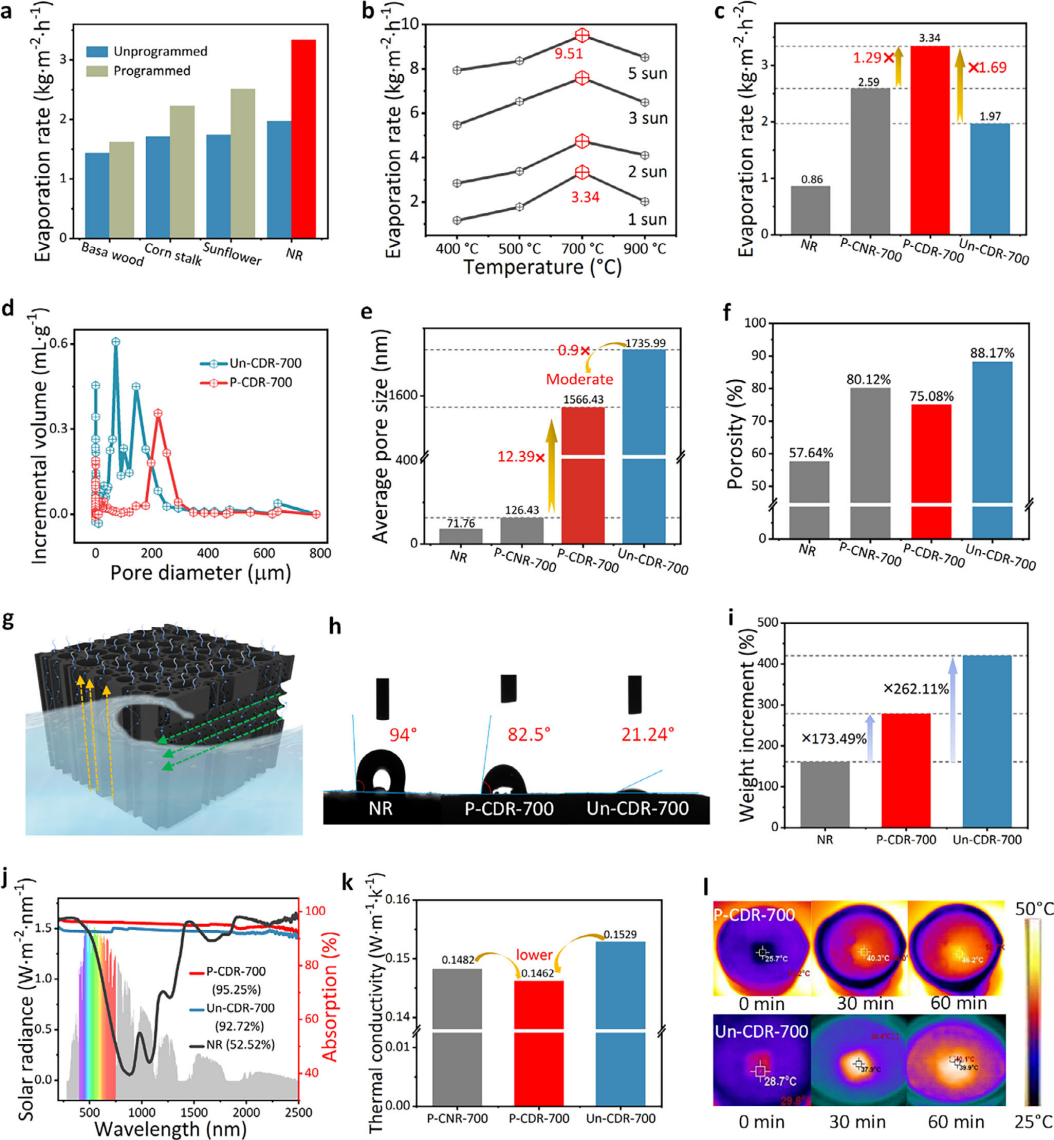
a) The evaporation rate of different biomass‐based evaporators prepared via programmed and unprogrammed carbonization, respectively. b) The evaporation rate of rattan‐based evaporator prepared at different final carbonization temperatures under different light intensities. c) The evaporation rate of rattan‐based evaporators with different treatments under 1 sun illumination. d) The pore size distribution of P‐CDR‐700 and Un‐CDR‐700. e) The average pore size and f) porosity of rattan‐based evaporators with different treatments. g) Water transport depending on micro‐channels. h) The water contact angle of NR, P‐CDR‐700, and Un‐CDR‐700. i) The mass change of NR, P‐CDR‐700, and Un‐CDR‐700 after being immersed in deionized water for 48 h. j) The optical absorption of NR, P‐CDR‐700, and Un‐CDR‐700. k) The thermal conductivity of rattan‐based evaporators with different treatments. l) The infrared thermal images of P‐CDR‐700 and Un‐CDR‐700 under 1 sun illumination for different time.

Natural rattan (NR) was composed primarily of large metaxylem vessels (200–360 µm in diameter), phloem vessels (20–80 µm) and slender protoplast vessels and sieve tubes (50–120 µm in diameter) (**Figure** **2**a‐1,c). Moreover, numerous pits, with heights of 0.5–1.5 µm and widths of 3–5 µm, were distributed along the cell walls (Figure 2a‐2,a‐3). These vascular channels and pits function as rapid conduits for water transport in both vertical and horizontal directions.^[^ ^43^ ^]^ After being treated with soft deliginification and programmed carbonization, the size of channels has some changes (Figure 2b‐1). For example, the phloem vessels exhibits an increased size to a range of 120–300 µm while the pits demonstrated a larger height of ≈3 µm and larger width of ≈10 µm (Figure 2b‐1,b‐3), which are also confirmed by the results of mercury intrusion porosimetry (MIP) (Figure 2c). Moreover, new pores were generated on intrafascicular parenchyma cells wall and highly loose skeletons were found on P‐CDR‐700 (Figure S4, Supporting Information). Figure 2c demonstrates MIP results of NR, rattan with programmed carbonization (labeled as P‐CNR‐700), and rattan with soft deliginification and programmed carbonization (labeled as P‐CDR‐700). Programmed carbonization was found to significantly contribute to the development of larger pores, broadening the macropore size range from 90–178 to 178–348 µm (Figure 2c). Larger pores and generation of new pores are beneficial for the improvement of water flux.^[^ ^44^, ^45^, ^46^, ^47^ ^]^

In the Fourier transform infrared (FT‐IR) spectra (Figure 2e,f), the characteristic peaks for lignin (1504 and 1423 cm^−1^) and hemicellulose (1730 and 1235 cm^−1^) were significantly diminished in the P‐CDR‐700 sample after treatment, confirming the effective removal of these components. NR, P‐CNR‐700, and P‐CDR‐700 display peaks near 3330, 3434, and 3440 cm^−1^, respectively, due to ─OH stretching vibrations. In P‐CDR‐700, peaks at 2935 and 1730 cm^−1^ were notably weakened. The peak near 1730 cm^−1^ diminished with rising temperature, likely from hemicellulose degradation. The C─O stretching vibration near 1031 cm^−1^ weakens due to cellulose and hemicellulose decomposition at high temperatures.^[^ ^42^, ^48^, ^49^ ^]^ In the X‐ray diffraction (XRD) spectra (Figure 2g), the diffraction peak at 22.1° in the P‐CDR‐700 sample was significantly broadened and its intensity was notably reduced, while in the P‐CNR‐700 sample, the intensity of this peak was lower compared to that of NR. These results indicate that the crystalline structure of cellulose further deteriorates during the carbonization process. Additionally, the diffraction peak observed at 42.6° in the P‐CDR‐700 sample corresponds to the (110) plane of graphite, indicating the formation of a graphitized structure during carbonization.^[^ ^50^ ^]^ The C 1s spectrum of P‐CDR‐700 can be deconvoluted into six peaks, corresponding to C─C (sp^2^), C─C (sp^3^), C─O, C═O, O─C═O, and the *π*–*π* ^*^ transition (Figure 2h; Figure S5, Supporting Information). The presence of the C─C (sp^2^) peak and the *π*–*π* ^*^ peak indicates the graphitization of rattan.^[^ ^51^ ^]^ The Raman spectroscopy was further used to characterize the graphitization. The ID/IG value of P‐CDR‐700 was 0.838, indicating that carbonization effectively promotes the graphitization of rattan (Figure S6, Supporting Information). The presence of graphitization is beneficial for enhancing photothermal conversion efficiency, thereby contributing to high evaporation efficiency.^[^ ^52^ ^]^

To evaluate the solar steam generation capability, the samples were wrapped in foam and floated on a beaker filled with water (Figure S7a, Supporting Information). Programmed and unprogrammed carbonization treatments were carried out on different biomass materials (including balsa wood, corn stalks, sunflower stalks, and rattan), and their evaporation rates under programmed and unprogrammed conditions were compared. The evaporation rates of the biomass evaporators treated with programmed treatment were significantly higher than those of the unprogrammed samples (**Figure** **3**a; Figure S7b, Supporting Information), confirming that programmed carbonization treatment has versatile applications for bulk biomass materials. Under a nitrogen atmosphere, 700 °C was determined to be optimal for achieving excellent evaporation rates (Figure 3b), and thus this temperature was used for carbonizing all experimental samples. Under 1 sun irradiation, P‐CDR‐700 achieved the highest rate at 3.34 kg·m^−2^·h^−1^, which is 1.69 times and 1.29 times higher than that of Un‐CDR‐700 and P‐CNR‐700, respectively (Figure 3c). This indicates that the programmed carbonization process contributes more significantly to enhancing the evaporation rates than the soft delignification process.

Therefore, the mechanism underlying the enhancement of evaporation by programmed carbonization has been studied. The MIP results reveal that the Un‐CDR‐700 exhibits a broad pore size distribution, mainly concentrated in three distinct pore size ranges: 50–90–90–178 µm. In contrast, the P‐CDR‐700 demonstrates a narrower pore size distribution, predominantly within (6–17 µm) and (178–348 µm) ranges (Figure 3d; Figure S8, Supporting Information). This is because programmed carbonization enables the gradual release of volatile species, which in situ etch the carbon framework, thereby preventing pore wall collapse and promoting the formation of hierarchical mesopores and macropores. It has been reported that such a synergistic pore structure could be effective for promoting high evaporation rate.^[^ ^53^ ^]^ Specifically, the small pores provide continuous capillary‐driven water transport, whereas the large pores provide low‐resistance channels for rapid vapor escape and highly water flux replenishment.^[^ ^53^ ^]^

**Figure 4:**
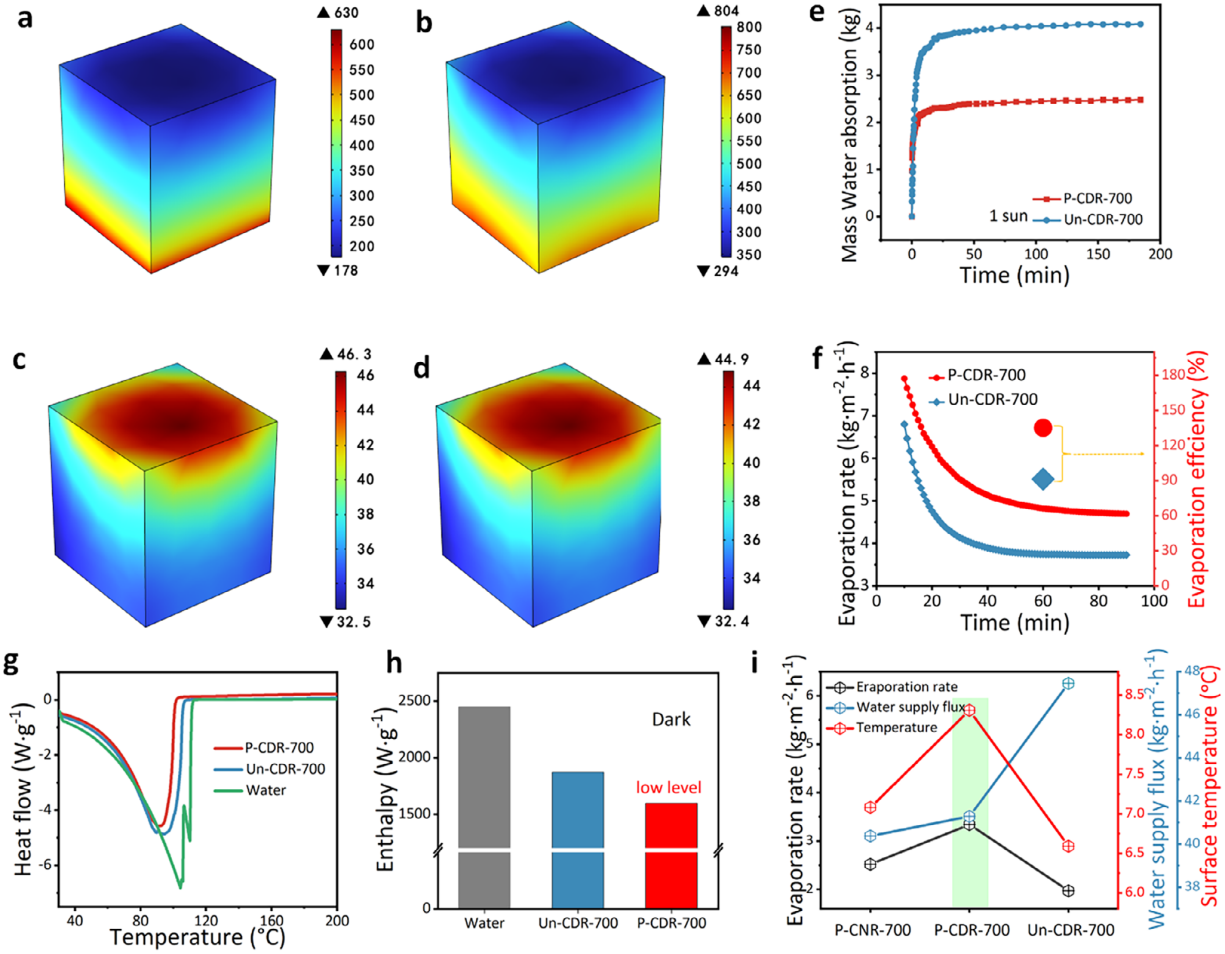
a,b) The water flow distributions and c,d) the temperature distributions for P‐CDR‐700 and Un‐CDR‐700, respectively. e) The water absorption ability of P‐CDR‐700 and Un‐CDR‐700. f) The simulated evaporation rate and efficiency. g) The DSC of pure water, pure water on P‐CDR‐700 and water on Un‐CDR‐700. h) The evaporation enthalpy of pure water, pure water on P‐CDR‐700 and Un‐CDR‐700. i) The surface temperature, evaporation rate, and water supply flux of P‐CNR‐700, P‐CDR‐700 and Un‐CDR‐700.

Crucially, however, increased water flux alone does not guarantee higher evaporation: excessive water supply can undermine heat localization by increasing heat loss and thickening the wet layer, which reduces the vaporization driving force.^[^ ^54^, ^55^, ^56^ ^]^ Surface wettability and water saturation tests were conducted to characterize the water transfer properties. Compared to natural rattan, P‐CDR‐700 and Un‐CDR‐700 exhibited water contact angles of 82.5° and 21.24°, respectively, both of which were less than 90°, indicating enhanced hydrophilicity (Figure 3g,h; Figure S9, Supporting Information). This can be attributed to the formation of larger pores, increased porosity and the introduction of hydrophilic groups after soft delignification and carbonization processing,^[^ ^57^, ^58^, ^59^ ^]^ as confirmed by SEM images, FT‐IR analysis, and MIP analysis (Figures 2a–c,e,f, 3d). Good hydrophilicity is beneficial for fast water transfer.^[^ ^60^ ^]^ Figure 3h shows that the unprogrammed sample (Un‐CDR‐700) exhibits superior surface hydrophilicity, which favors water transport and may result in a faster water transfer rate than P‐CDR‐700. The water saturation test demonstrates that after 48 h of immersion (Figure 3i), the weights of P‐CDR‐700 and Un‐CDR‐700 were 173.49% and 262.11% of the weight of the NR sample, respectively. It can be found that both P‐CDR‐700 and Un‐CDR‐700 samples exhibit great water transport properties to ensure sufficient water supply for continuous solar‐driven interfacial evaporation. The Un‐CDR‐700 demonstrates a more significant improvement in this regard. However, excessive water saturation in the pores or on the surface of evaporators can lead to unavoidable heat loss, thereby impairing the evaporation rate.^[^ ^61^, ^62^ ^]^ Given that interfacial temperature is influenced by multiple factors such as thermal management, COMSOL simulations were employed to decouple these effects, enabling a focused investigation of water transport as a single factor in governing heat localization. The results show that the reduced water supply in P‐CDR‐700 leads to a higher interfacial evaporation temperature (**Figure** **4**a–d), suggesting that optimized water transport and heat localization are achieved compared with the unprogrammed sample. More details will be discussed in the following section. The average pore diameter calculated from pore size and pore volume was 1566 nm for the programmed P‐CDR‐700, smaller than that of the unprogrammed sample (1736 nm), but still much larger than that of NR (126 nm) (Figure 3e). Moreover, the porosity of P‐CDR‐700 (75%) was lower than that of Un‐CDR‐700 (88%) (Figure 3f). However, but remains significantly higher than that of natural rattan. This intermediate pore size means that P‐CDR‐700 provides sufficient water replenishment without causing oversupply, thereby maintaining an optimal water transport and heat localization balance.

The effect of porous structure on thermal management was also investigated. P‐CDR‐700 exhibited broadband light absorption of up to ≈95%, which is slightly higher than that of Un‐CDR‐700 (≈93%) (Figure 3j). Moreover, P‐CDR‐700 showed a marginally lower thermal conductivity (0.1462 W m^−1^k^−1^) compared to Un‐CDR‐700 (0.1529 W m^−1^ k^−1^) (Figure 3k). The surface temperature measurements confirm the excellent heat localization capability of P‐CDR‐700 (Figure 3l). Within one hour, its surface temperature escalated to 46.2 °C, in contrast to 40.1 °C for the Un‐CDR‐700, indicating better heat localization on the evaporation interface. Although the increase in light absorption and the decrease in thermal conductivity are beneficial for heat localization,^[^ ^63^, ^64^ ^]^ the improvements are relatively small, and thus their overall contribution to heat confinement is limited.

The heat localization is also determined by water transport.^[^ ^56^ ^]^ The water supply of P‐CDR‐700 was slightly lower than that of Un‐CDR‐700. We hypothesized that this reduced supply would facilitate a more optimal balance between water transport and heat localization. COMSOL simulations, which couple heat transfer and fluid flow in porous media within a stationary solver, were employed to clarify the contributions of the optimized water transport to the heat localization (details seen Note S2, Supporting Information). In the simulations, the density, specific heat capacity, and thermal conductivity of both the programmed and unprogrammed samples, which are highly dependent on microstructure, were assigned based on experimental measurements. Solar illumination, and photothermal conversion were fixed at identical levels in all simulations to decouple their effects. The simulated P‐CDR‐700 exhibited lower water content but a higher surface temperature compared to the simulated Un‐CDR‐700 (Figure 4a–d), which is consistent with the experimental observation (Figure 4e,3l). The simulation shows that the programmed P‐CDR‐700 achieves higher evaporation rate compared with that of the unprogrammed sample (Figure 4f), which is in good agreement with the experimental results (Figure 4i), and highly agrees with the experiments confirming the contribution of programmed porous structures to optimize the balance between water transport and heat localization and thus boost evaporation rate.

The above discussion illustrates that the programmed structure plays a crucial role in balancing water and heat. In addition, it also contributes to a reduction in the evaporation enthalpy. Differential scanning calorimetry (DSC) analysis revealed that the equivalent enthalpies of Un‐CDR‐700 and P‐CDR‐700 were 1927, 1610 J g^−1,^ respectively (Figure 4g). The estimated enthalpy under dark conditions were consistent with the results of DSC (Figure 4h). Programmed sample with lower evaporation enthalpy could effectively reduce the energy required for evaporation. The reduced evaporation enthalpy of the programmed sample can be attributed to its pore structure. MIP analysis reveals that the programmed P‐CDR‐700 possesses a bimodal hierarchical pore structure, and in the small‐pore region, its pore sizes (6–17 µm) are markedly smaller than those of the unprogrammed Un‐CDR‐700 (50–90 µm) (Figure 2c). Smaller pores promote the formation of interfacial water with a less extensive hydrogen‐bond network, thereby lowering the energy required for vaporization.^[^ ^65^ ^]^

In summary, the high evaporation rate primarily arises from the synergistic structure, which enables an optimal water transport and thermal localization balance, facilitates rapid vapor escape, and reduces the effective evaporation enthalpy.

**Figure 5:**
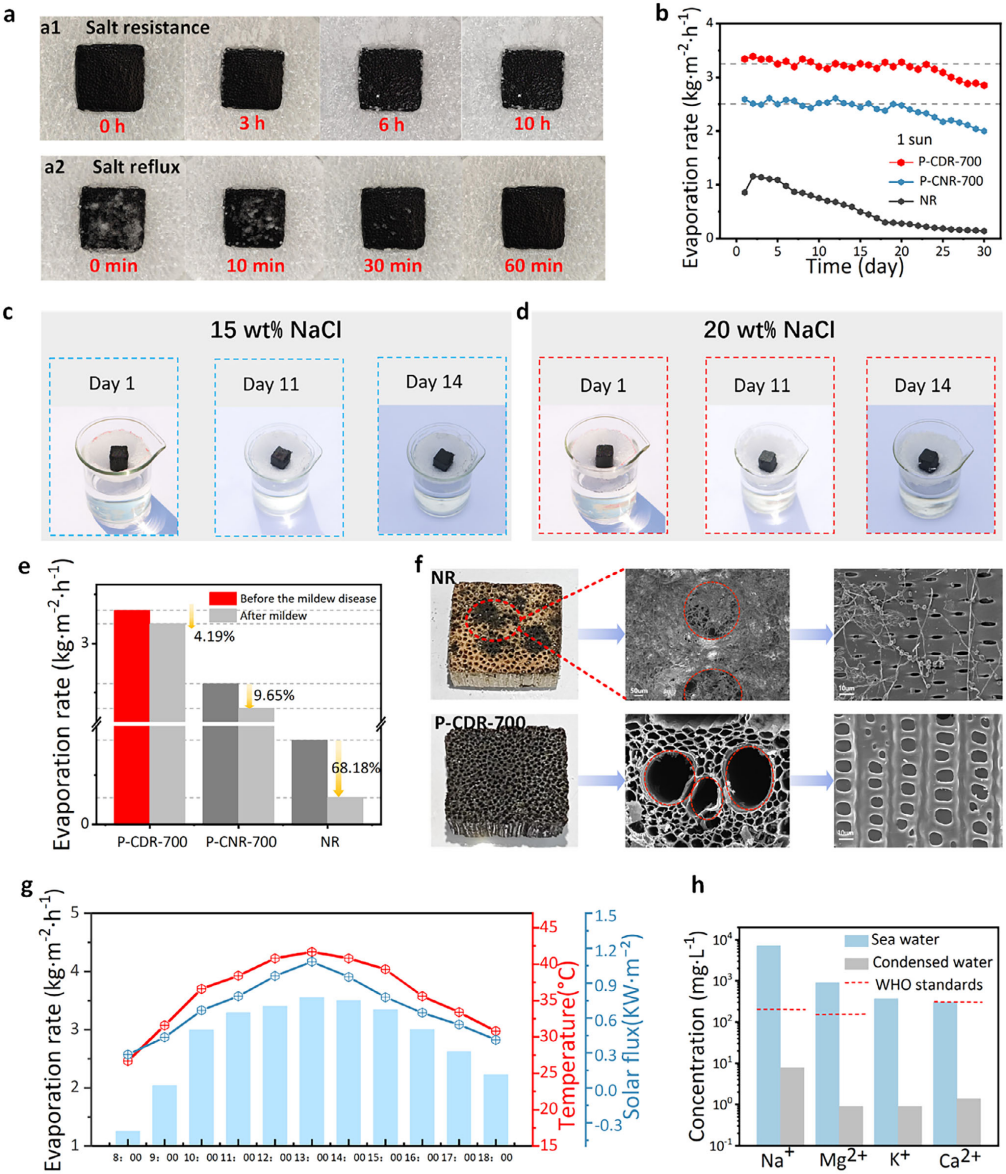
a) The salt‐resistant and self‐cleaning capability of the P‐CDR‐700 (a1 for simulated seawater desalination, a2 for artificial added salt under simulated sunlight irradiation). b) The durability of NR, P‐CNR‐700, and P‐CDR‐700 under 1 sun irradiation. c,d) 14‐day outdoor evaporation in 15 and 20 wt.% NaCl solution, Chengdu, China, daily photographs at 9:30 a.m. e) The evaporation rate of different rattan‐based evaporators before and after contamination. f) The change in morphology of NR and P‐CDR‐700 after the same fungal contamination test. g) The real‐time monitoring of evaporation mass, ambient temperature, and solar flux of P‐CDR‐700 during outdoor tests in Chengdu. h) The concentration of four primary seawater ions (Na^+^, Mg^2+^, Ca^2+^, and K^+^) before and after desalination using P‐CDR‐700.

The long‐term application of biomass‐based BBSEs in seawater desalination is important for practical application.^[^ ^66^, ^67^ ^]^ The evaporation rate of P‐CDR‐700 decreased with salt concentration increasing (Figures S10 and S11a,b, Supporting Information). However, it remained 1.76 kg m^−2^ h^−1^ at 20 wt.% NaCl solution, outperforming that of NR and most other reported BBSEs (Figure S11c, Supporting Information). Moreover, after 10 h of sunlight exposure in simulated seawater, only a negligible amount of NaCl crystals formed on the surface of P‐CDR‐700 (**Figure** **5**a‐1). Upon the artificially added of NaCl crystals, complete dissolution occurred within 60 min, regenerating a clean evaporation surface (Figure 5a‐2). Self‐cleaning property from salt reflux is beneficial for salt resistance under long‐term application.^[^ ^57^ ^]^ Salt resistance property was evaluated by continuous outdoor evaporation in NaCl solutions with concentrations of 3.5, 5, 10, 15, and 20 wt.%. The results showed that no crystallization was observed at 3.5–15 wt.% NaCl, with only negligible edge crystallization at 20 wt.% over 14 consecutive days (Figure 5c,d; Figures S12–S16, Supporting Information). Notably, the salt resistance should extend well beyond 14 days, but the tests were interrupted by rainfall on the 14th day. The excellent salt resistance property can be attributed to its bimodal hierarchical pore structure (Figure 2c). In bimodal hierarchical structures, the small pores (6–17 µm) serve as capillaries that continuously draw water to the evaporation surface, while the large pores (178–348 µm) provide low‐resistance channels for liquid transport. When the evaporation rate decreases, the capillary action still supplies fresh water, diluting the concentrated brine at the interface and allowing excess salt to diffuse or flow back to the bulk water through the large channels, thereby preventing persistent salt crystallization.^[^ ^68^ ^]^ Furthermore, to monitor the stability of the evaporation rate, a cyclic test was conducted for 30 days, with each day consisting of 2 h of continuous evaporation under 1 sun illumination followed by 22 h of drying. The results show that P‐CDR‐700 demonstrated minimal change in evaporation rate over 23 days, while natural rattan‐based evaporators became unstable after 20 and 5 days, respectively (Figure 5b).

Long‐term application not only depends on good salt resistance but also mold resistance. Mold growth on the evaporator not only impedes water transport but also significantly reduces the effective area for light absorption and increases vapor diffusion resistance, resulting in a substantial decline in the evaporation rate.^[^ ^15^ ^]^ As shown in Figure 5e, the evaporation rate of NR decreased significantly by 68.18% after prolonged immersion in river water. In contrast, P‐CDR‐700 and P‐CNR‐700 exhibited only minimal reductions of 4.19% and 9.65%, respectively. Both the carbonization and delignification processes effectively enhanced mold resistance by removing nutrients essential for fungal growth, such as starch (Figure 2d). SEM images further confirm the superior mold resistance, as NR was extensively covered with fungal hyphae, whereas no fungal accumulation was observed on the surface of P‐CDR‐700 under the same fungal contamination test conditions (Figure 5f; Figure S17, Supporting Information).

In practical applications, P‐CDR‐700 has been validated to deliver outstanding water purification in both seawater desalination and wastewater treatment, highlighting its potential for real‐world deployment. When placed in a condensate collector for outdoor desalination, its evaporation rate increased with temperature and irradiance, reaching 3.55 kg m^−2^ h^−1^ between 12:00 and 13:00 (Figure 5g). A high evaporation rate (> 3 kg·m^−2^·h^−1^) was maintained for most of the daylight hours. Desalination was assessed by measuring key metal ions (Na^+^, Mg^2+^, K^+^, and Ca^2+^) in seawater.^[^ ^69^ ^]^ After evaporation, their concentrations dropped significantly, meeting WHO drinking water standards (Figure 5h). Moreover, the water purification function was also effective for real food‐waste swill and dye‐containing wastewater (e.g., methylene blue and methyl orange solutions) (Figure 5h; Figures S18 and S19, Supporting Information). Furthermore, modular scalability was demonstrated by assembling a 5 × 5 array of P‐CDR‐700 evaporators, which produced ≈24.49 L of freshwater during outdoor operation from 9:00 to 18:00 (Figure S20, Supporting Information), highlighting its promise for real‐world application.

## 3 Conclusion

In summary, an efficient and versatile approach to high‐performance biomass‐based solar evaporators has been developed via programmed carbonization. The evaporation rate of P‐CDR‐700 under 1 sun illustration reaches 3.34 kg·m^−2^·h^−1^, which is 1.69 times higher than that of the unprogrammed carbonization treatment (Un‐CDR‐700). This performance outperforms most existing carbonized biomass‐based evaporators and represents the best among rattan‐based evaporators. Critically, P‐CDR‐700 achieves exceptional salt durability: it withstands continuous outdoor evaporation for over 14 days while processing high‐salinity brine (20 wt.% NaCl), without noticeable salt accumulation or microbial growth. Outdoor experiments further confirm its stable daily fresh water production (≈24.49 L) with high quality that meets WHO drinking standards. Both experimental and simulation results confirm that the engineered bimodal hierarchical pore structure of P‐CDR‐700 enables an optimized balance between water transport and thermal localization, facilitates rapid vapor escape, and reduces the effective evaporation enthalpy, thereby enhancing the evaporation rate. This approach demonstrates versatile application towards biomass, including but not limited to balsa wood, corn stalk, sunflower, and reed cane. The work provides valuable insights for the further development of long‐term, efficient seawater desalination systems.

## 4 Experimental Section

The details were shown in the Supporting Information.

## Conflict of Interest

The authors declare no conflict of interest.

## Supporting information



Supporting Information

## Data Availability

The data that support the findings of this study are available in the supplementary material of this article.
