# Supplementary material for: Rattan‐Based Solar Evaporator Hits 3.34 Kg·M−2·h−1 and Long‐Term Salt Resistance via Programmed Carbonization
Source: Adv Sci (Weinh). 2025 Oct 24;13(1):e11782. doi: 10.1002/advs.202511782 (PMC12766989; doi:10.1002/advs.202511782)
Supplement: Supplementary file 1 — Supporting Information [file ADVS-13-e11782-s001.doc]

**Supplementary Materials**

**Rattan-Based Solar Evaporator Hits 3.34 kg·m^-2^·h^-1^ and Long-Term Salt Resistance via Programmed Carbonization**

Shanshan Jia*^a,b,1^*, Xiaqing Qin*^a,1^*, Shijie Dai*^a^*, Yan Qing*^c^,**, Jianwu Dai^d^, Shaobo Zhang*^a^*, Hui Xiao*^a^*, Yuzhu Chen*^a^*, Jinqiu Qi*^a^*, Yongqian Yang*^a^*, Yao Lu^e^*,**, Zhiping Su*^a^,**, Yong Zhuo^f^, De Wu^e^,*

*^a^ College of Forestry, Sichuan Agricultural University, Chengdu 611130, P.R. China*

*^b^ Forest Ecology and Conservation in the Upper Reaches of the Yangtze River Key Laboratory of Sichuan Province. Chengdu 610000, P. R. China*

*^c^ College of Materials Science and Engineering, Central South University of Forestry and Technology, Changsha 410004, P. R. China*

*^d^ College of Mechanical and Electrical Engineering, Sichuan Agricultural University, Ya'an, China*

*^e^ Department of Chemistry, School of Physical and Chemical Sciences, Queen Mary University of London, London, E1 4NS UK*

*^f^ Animal Nutrition Institute, Sichuan Agricultural University, Chengdu 611130, P.R. China*

Corresponding Authors

*Email address:* [*qingyan0429@163.com*](mailto:qingyan0429@163.com)*, yao.lu@qmul.ac.uk, suzp@sicau.edu.cn, wude@sicau.edu.cn.*

**1. Experiment and Methods**

**1.1 Materials**

Rattan (Indonesian rattan) was purchased from Foshan Yirui Shang'an Industry and Trade Co., Ltd. and stored at room temperature. Balsa wood was purchased from Sichuan Babilu Co. ltd, corn stalk, sunflower and corn stalk were purchased from local market. Sodium sulfite (Na_2_SO_3_) and sodium hydroxide (NaOH) were obtained from Chengdu Kelong Chemical Co., Ltd., hydrogen peroxide (H_2_O_2_) was Purchased from Sichuan Xilong Scientific Co., Ltd., and methylene blue (MB), methylene orange (MO), and copper sulfate pentahydrate (CuSO_4_·5H_2_O) were supplied by Aladdin Biochem Technology Co., Ltd. (Shanghai, China). All chemicals were of analytical grade.

**1.2 Fabrication of P-CDR-700、Un-CDR-700 and P-CNR-700**

A 15mm×15mm×15mm rattan cube was treated in a 2.5 mol·L^-1^ NaOH and 0.4 mol·L^-1^ Na_2_SO_3_ mix for 12 h, washed, then heated in 2.5 mol·L^-1^ H_2_O_2_ for 3h, washed again, and freeze-dried at -70 °C to obtain delignified rattan (DR). DR underwent programmed heating (10 °C·min^-1^) to 350 °C for 40 min, 600 °C for 60 min, and carbonized at 700 °C for 4h to yield P-CDR-700. This heating program was set according to the result of TG (Figure S1). Natural rattan of the same size was carbonized at 700 °C for 4 h to get P-CNR-700. Un-CDR-700 was obtained by directly heating DR to 700 °C for 4h without programming. Other biomass materials, such as wood and sunflower, were also treated with these two differences carbonization process when preparing solar evaporators.

**1.3 Characterization**

The samples' microscopic morphology was examined using a field emission scanning electron microscope (Gemini 300). FT-IR (Nicolet iS20) analyzed chemical composition and bonding. Pore size distribution was measured with an automated mercury intrusion porosimeter (MIP, AutoPore IV 9510). XRD (Ultima IV) was used for structural analysis. Elemental composition was investigated via XPS (K-Alpha). An infrared thermal imager (UNI-T UTi220A) captured thermal images, and absorbance was measured using a UV-Vis-NIR spectrometer (UV-3600 iPlus) from 250 nm to 2500 nm.

1.4 Solar Steam Generation Test

The sample was placed in a beaker containing 50ml of pure water or seawater, with a layer of hydrophobic polyethylene foam covering the space between the rattan sample and the liquid in the beaker to minimize experimental errors arising from additional exposure to the air-liquid interface ^[33]^. A xenon lamp equipped with optical components (AM 1.5) was employed to evaluate the solar steam generation capability. During the experiments, the weight change of the evaporation system was measured using an electronic balance, while the temperatures of the sample and the water were monitored with an infrared camera. All experiments were conducted at a temperature of (25±5) °C and an ambient humidity of (50±10%). The evaporation rate $\left(\text{υ}\right)$ and evaporation efficiency $\left(\eta\right)$ were calculated using Equations 1 and 2, respectively ^[34]^.

$$\text{υ (kg}\times\text{m}^{\text{-2}}\times\text{h}^{\text{-1}}\text{)}=\frac{\Delta\text{m}}{s\times\Delta t}$$

$$\eta(\%)=\frac{\text{υ}\times\Delta H\omega}{Copt\times qi}\times100\text{\%}$$

Herein, $\Delta\text{m}$ (kg) represents the mass change during water evaporation, *s* (m^2^) denotes the evaporation surface area, and $\Delta t$ (h) represents the corresponding evaporation time. $\Delta H\omega$ (J·g^−1^) refers to the enthalpy of water evaporation, Copt is the optical concentration, and $qi$ is 1 kW·m^−2^. To ensure the validity of the calculated efficiency, the natural evaporation rate of the rattan sample under dark conditions must be subtracted from the measured evaporation rate under solar irradiation.

The evaporation rate of P-CDR-700 was obtained by subtracting dark environment rate to exclude natural evaporation effects.

**2. COMSOL Simulation Supplementary Note**

The heat transfer and moisture transfer module of COMSOL Multiphysics 6.2 numerically simulates the heat transfer and moisture transfer process during evaporation of different samples. Here, the input solar energy of one sun, the environment and water temperature of 20 °C (293.15K), and the surface temperature are used as boundary conditions. The water-related parameters are directly taken from the COMSOL Multiphysics material library. The geometric shape of the sample was modeled by independent software. In the simulation process, the natural convection heat transfer coefficient is set to 5 W·m^-2^·K^-1^. The density, specific heat capacity and thermal conductivity of the two samples are 0.288 g·cm^-3^, 1.97 J g^-1^·K^-1^ and 0.1462 W·m^-1^·K^-1^ and 0.1419 g·cm^-3^, 1.65 J·g^-1^·K^-1^ and 0.1529 W·m^-1^·K^-1^, respectively. In the case of these boundaries and parameter matching, the temperature distribution in the COMSOL model is described by the following equation:

$$Q+Q_{evap}=\rho C_{P}\frac{\partial V}{\partial t}+\rho C_{P}u\cdot\nabla T+\nabla\cdot q$$

$$q=-k\nabla T$$

1. $Q$ is the heat input from solar radiation;
2. $\rho$is the mass density of the substance;
3. $C_{P}$is the specific heat capacity of the substance;
4. $k$ is the thermal conductivity of the material;
5. $T$ is the ambient temperature;
6. $u$ is the fluid velocity of the water medium;
7. $Q_{evap}$ is the latent heat source of evaporation.

Water transport is described by the following equations:

1. is the total moisture content, including liquid water and water vapor;
2. is relative humidity;
3. $\mu_{g}$ is the velocity field of moist air;
4. $\omega_{v}$ is the mass fraction of water vapor in moist air;
5. $g{}_{\omega}$ is the diffusion flux of moist air;
6. $\mu_{l}$ is the velocity field of liquid water;
7. $g_{lc}$ is the capillary flux of liquid water, expressed by the capillary pressure gradient;
8. $G$ is the water source term.

The theoretical simulation was carried out in the transient analysis mode using COMSOL Multiphysics 6.2. The ambient temperature is set to 293.15K, and the thermal convection between the evaporation surface and the air is corrected by Newton 's Cooling Law. More details see Table S2-S3.

1. **Figures**

**
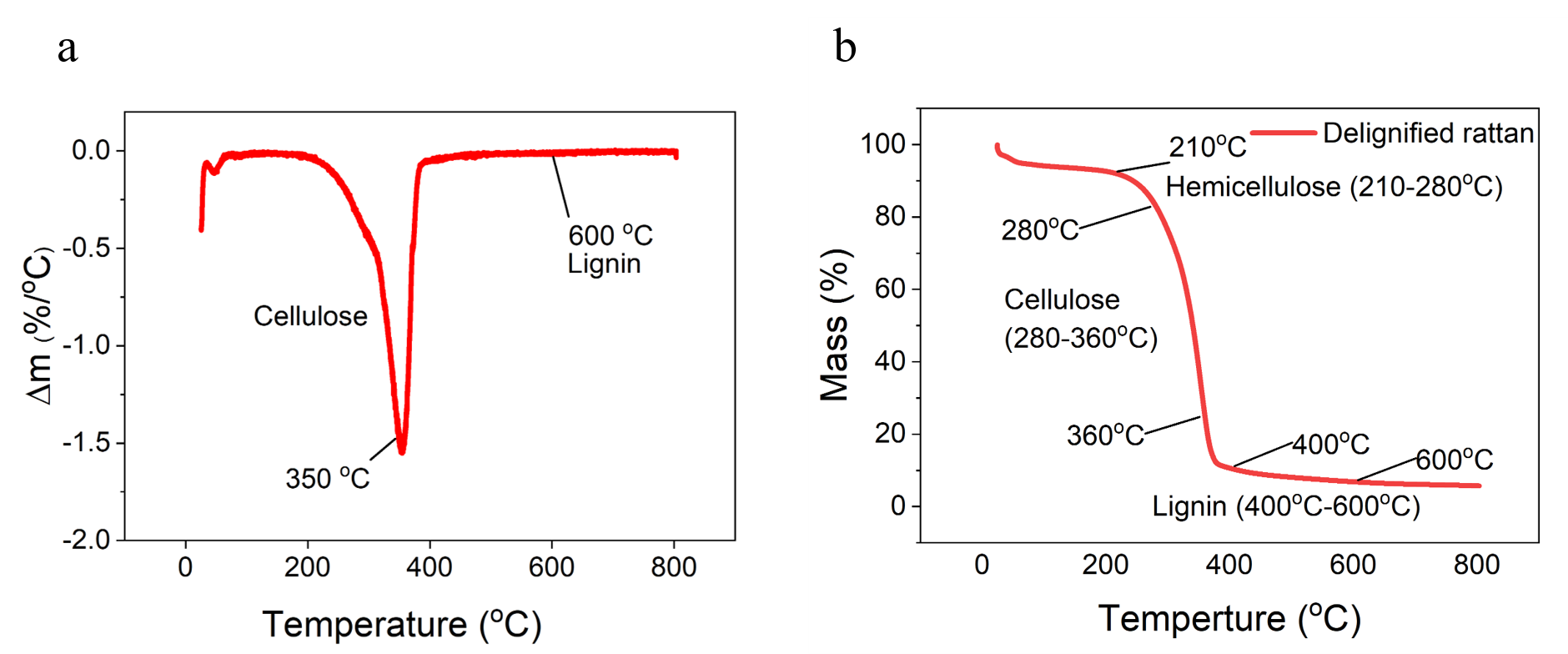
**

**Figure S1.** TG/DTG guided heat program ( holding at 350 °C and 600 °C during carbonization).


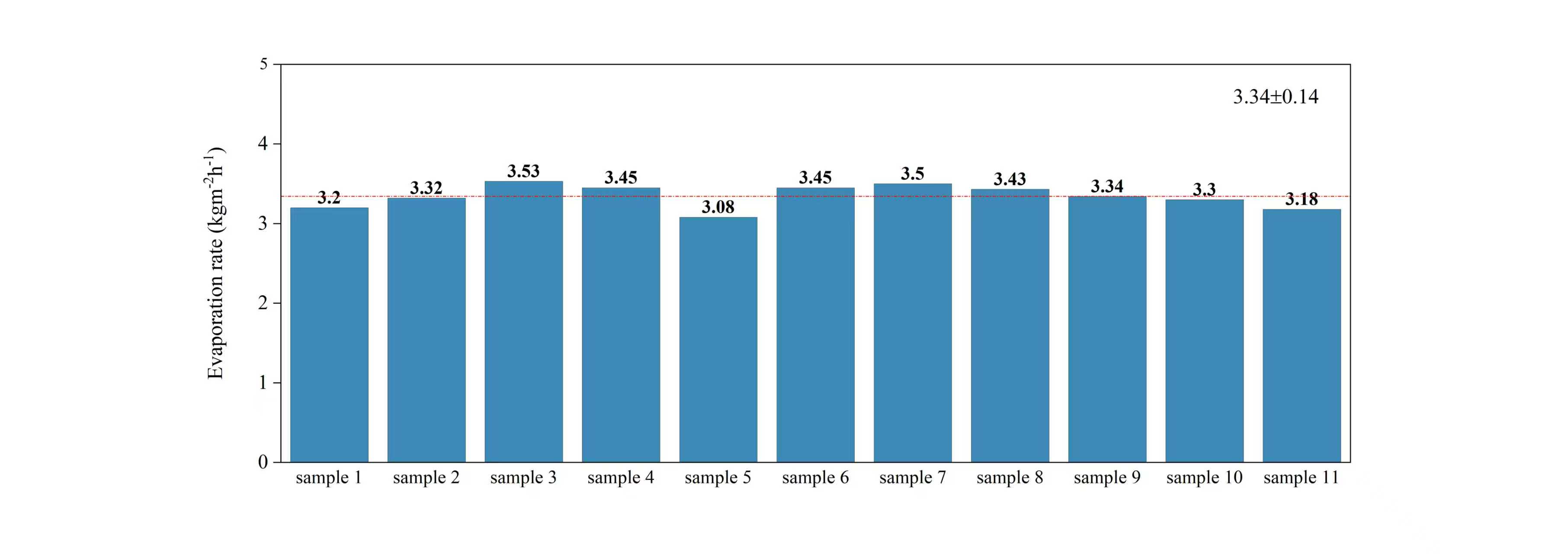


**Figure S2**. Evaporation rates of 11 P-CDR-700 samples.


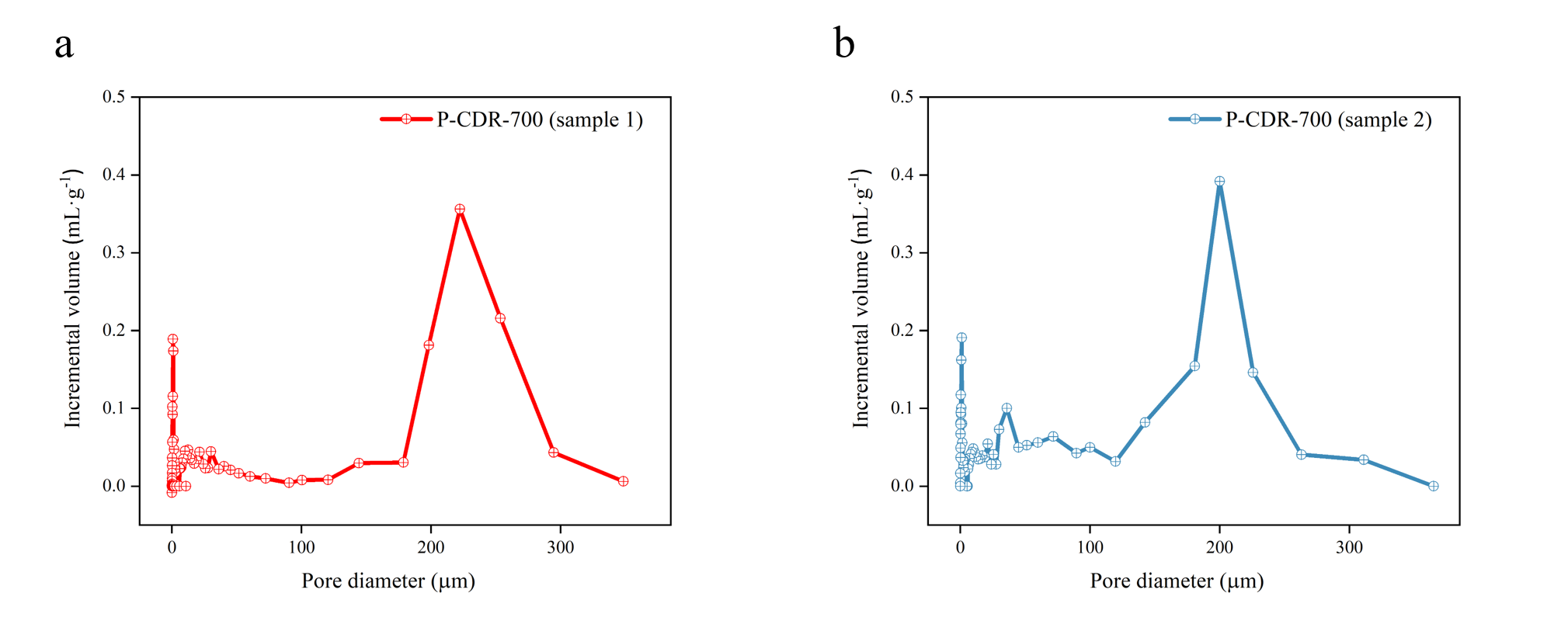


**Figure S3**. Pore size distribution of randomly selected programmed samples.

**
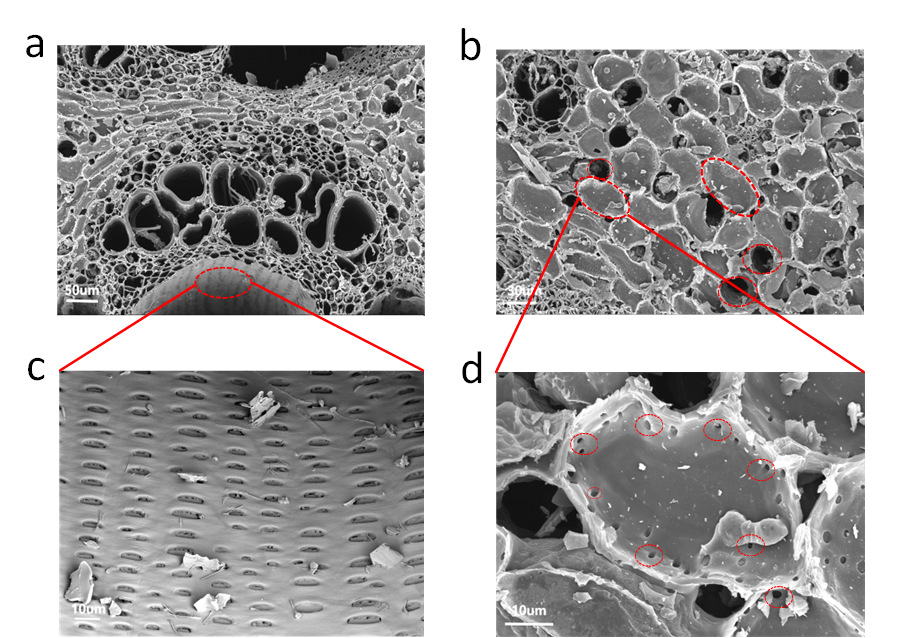
**

**Figure S4.** Cross-sectional and internal morphology of P-CDR-700. a, b, d) SEM images of cross-sections. c) SEM image showing the inner wall of the conduit.


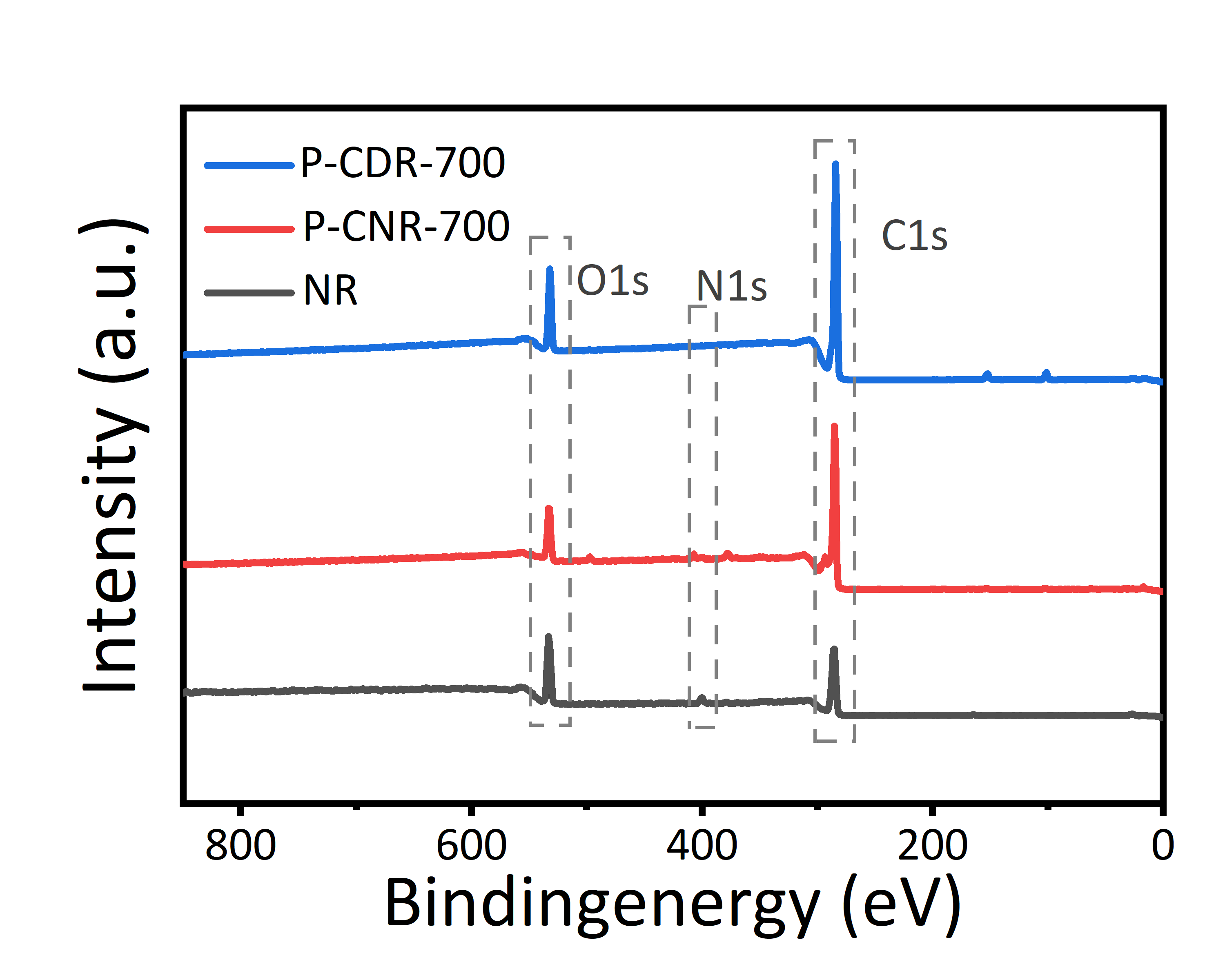


**Figure S5.** X-ray photoelectron spectroscopy (XPS) survey spectra of C, N, and O elements in NR, CNR-700, and CDR-700.


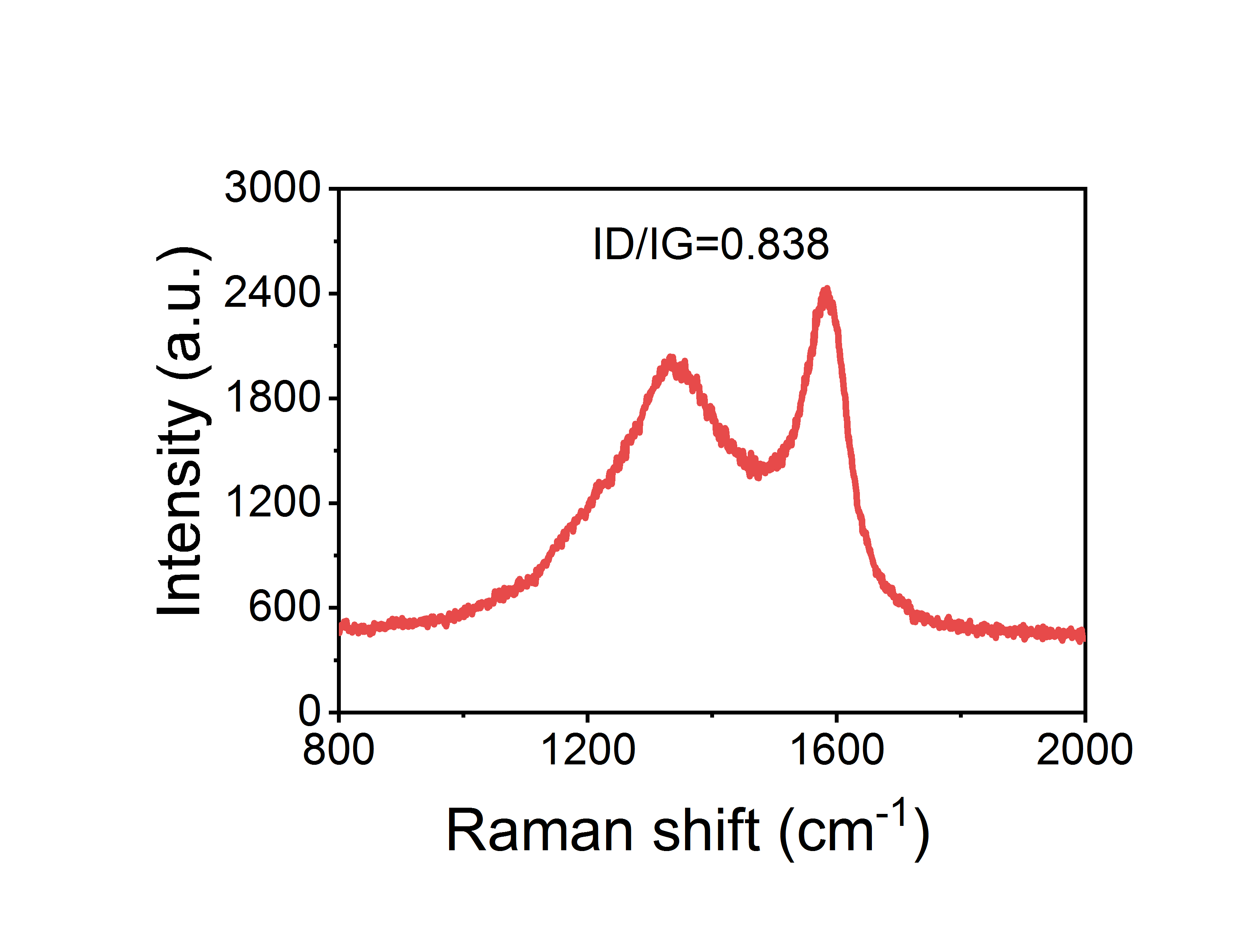


**Figure S6.** The Raman spectroscopy of P-CDR-700.


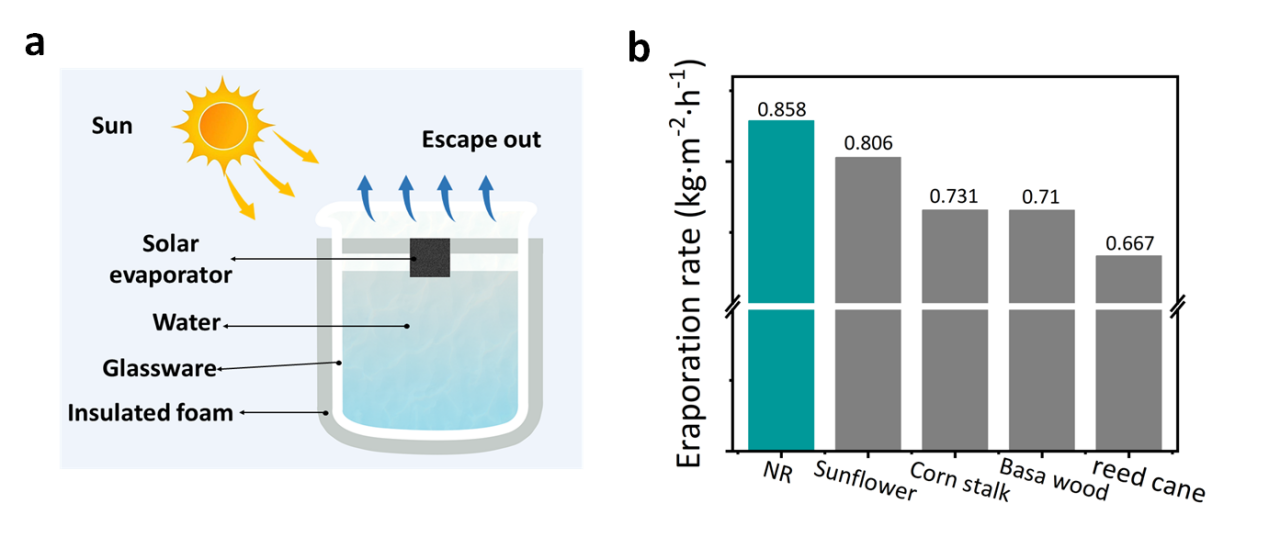


**Figure S7.** (a) Schematic of the solar-driven interfacial evaporation device; (b) Comparison of evaporation rates among different natural biomass-derived materials.


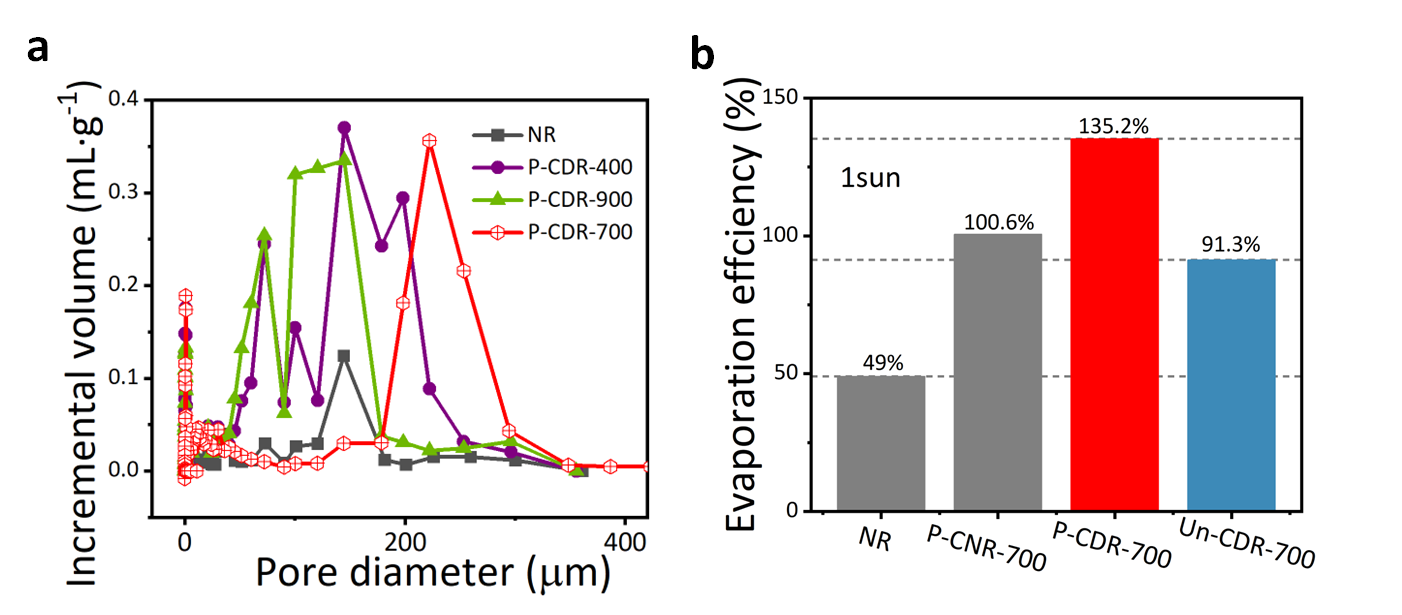


**Figure S8.** (a) Pore size distributions of temperature-varied carbonized rattan evaporators; (b) Evaporation efficiency comparison under 1 sun for differently processed rattan evaporators.

Excessively high or low carbonization temperatures can affect the pore structure inside the evaporator. An excessively high temperature can cause partial collapse of the pore walls, reduce the effective specific surface area and thereby degraded the evaporation performance of the rattan-based evaporator. However, P-CDR-700 possesses the optimal bimodal hierarchical pore structure, which is beneficial for boosting evaporation rate.


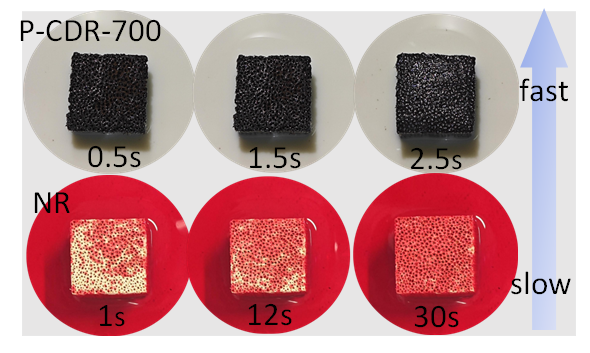


**Figure S9.** Water transport performance comparison between P-CDR-700 and NR.

**
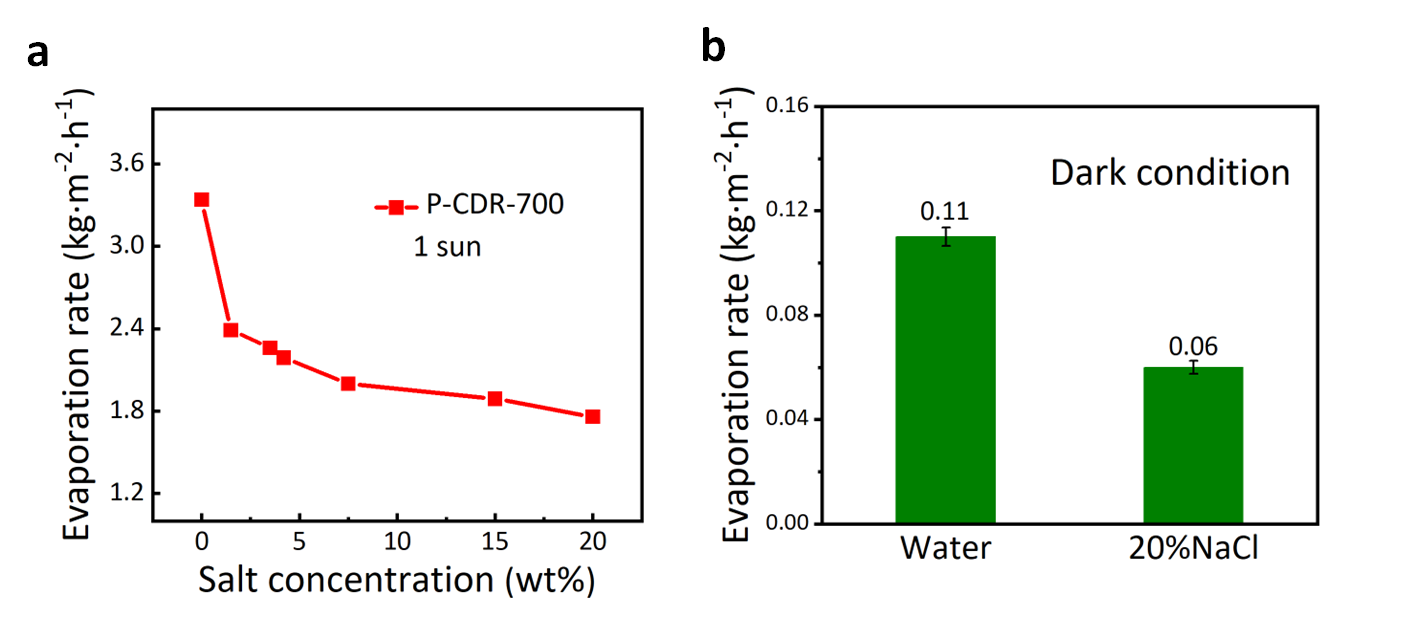
**

**Figure S10.** (a) Concentration-dependent evaporation rates of P-CDR-700 in NaCl solutions; (b) Dark condition evaporation performance: pure water vs. P-CDR-700.


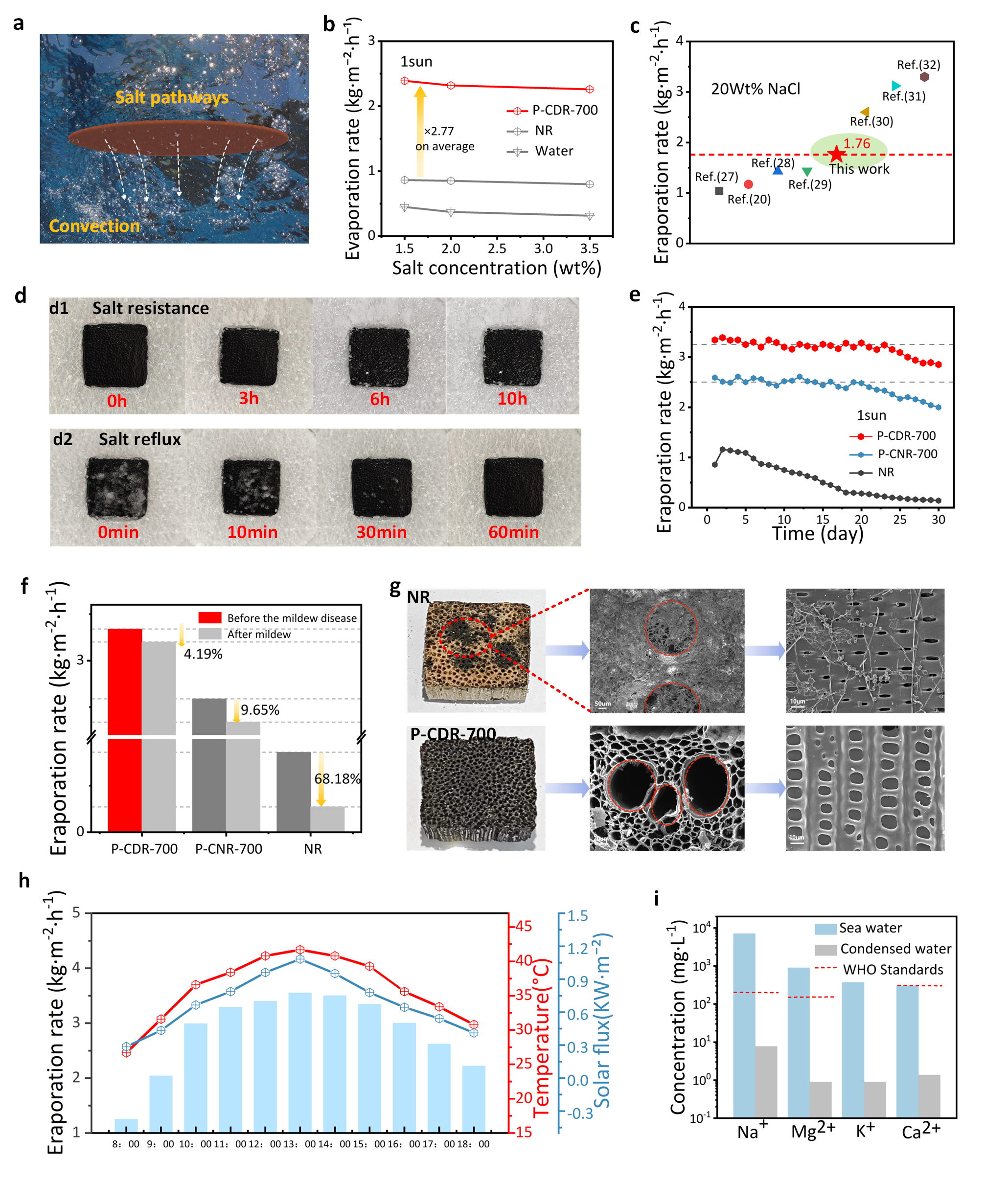


**Figure S11. a** Water transport route within the rattan-based evaporator. **b** Evaporation rate line charts comparing P-CDR-700 and NR at varying salt concentrations. **c** Comparison of evaporation rates between our evaporator and conventional models in 20 wt% saline solution (detailed references provided in Supplementary Table 1).


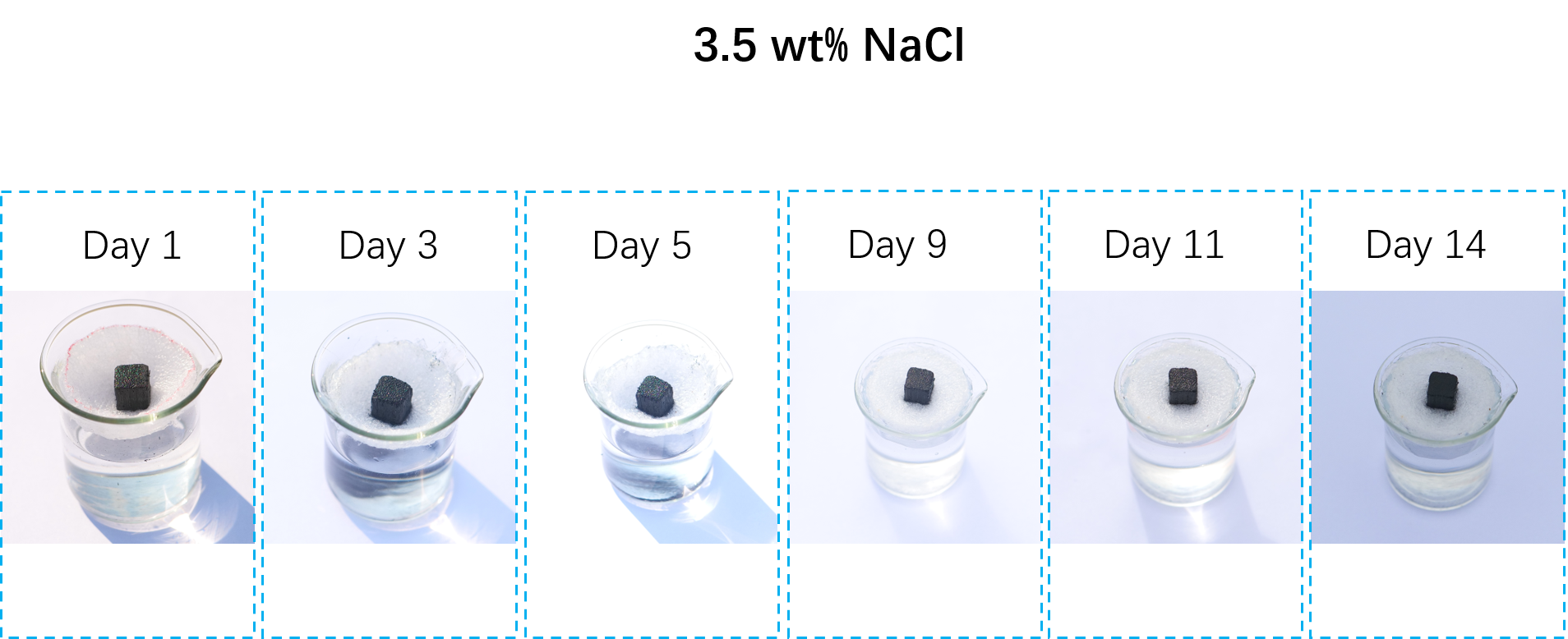


**Figure S12.** 14-day outdoor evaporation in 3.5 wt% NaCl brine, Chengdu, China; daily photographs at 9:30 a.m.


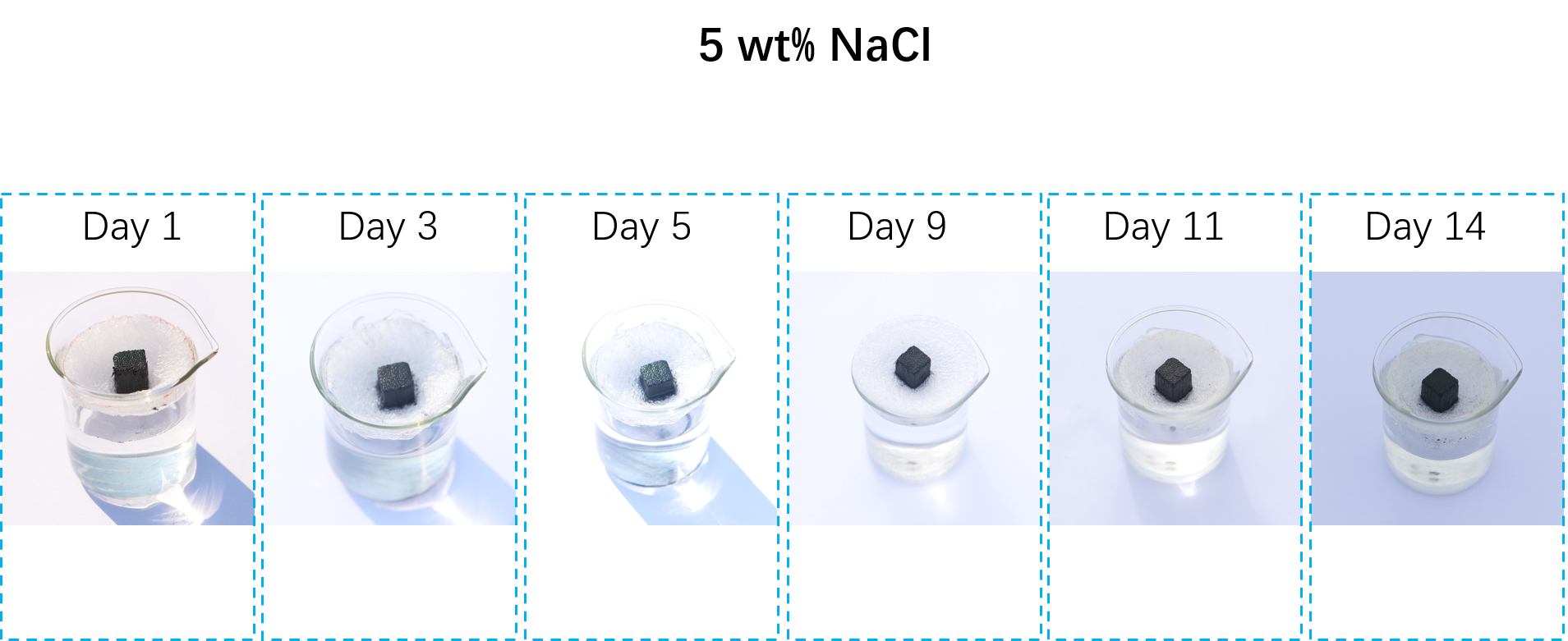


**Figure S13.** 14-day outdoor evaporation in 5 wt% NaCl brine, Chengdu, China; daily photographs at 9:30 a.m.


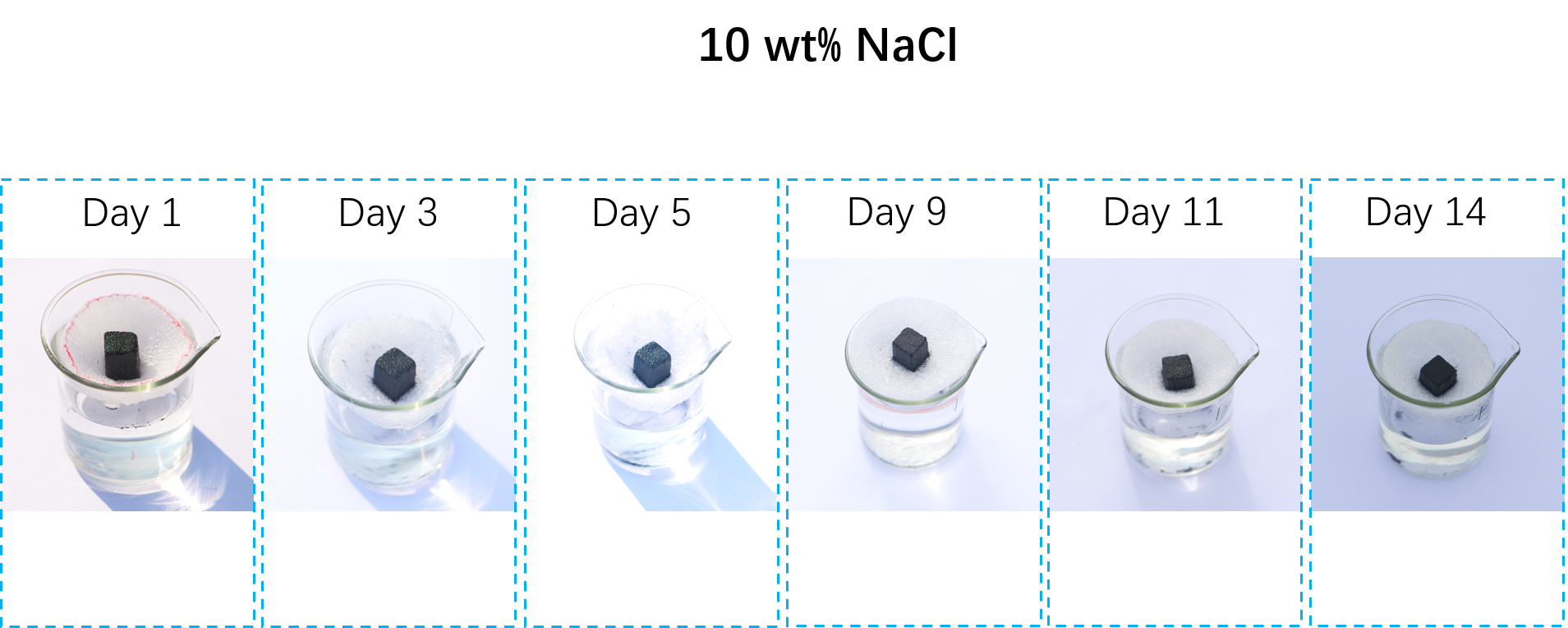


**Figure S14.** 14-day outdoor evaporation in 10 wt% NaCl brine, Chengdu, China; daily photographs at 9:30 a.m.


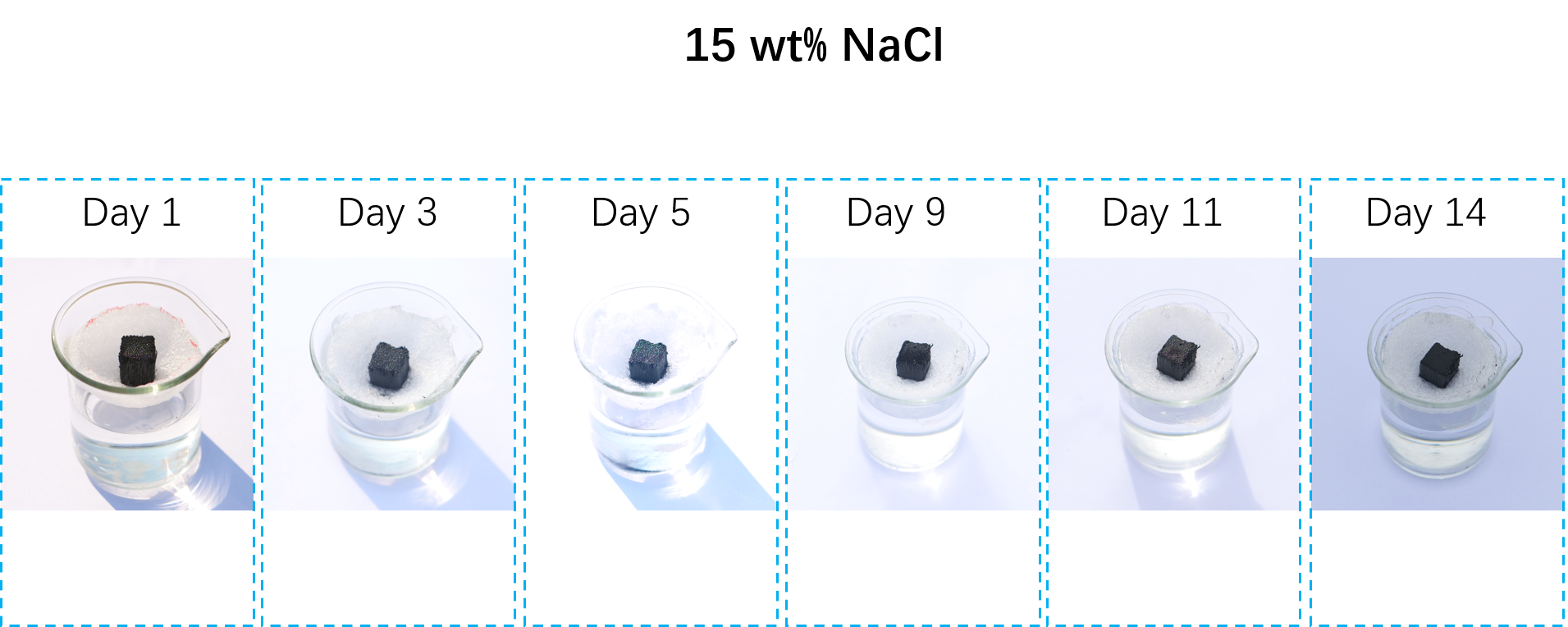


**Figure S15.** 14-day outdoor evaporation in 15 wt% NaCl brine, Chengdu, China; daily photographs at 9:30 a.m.


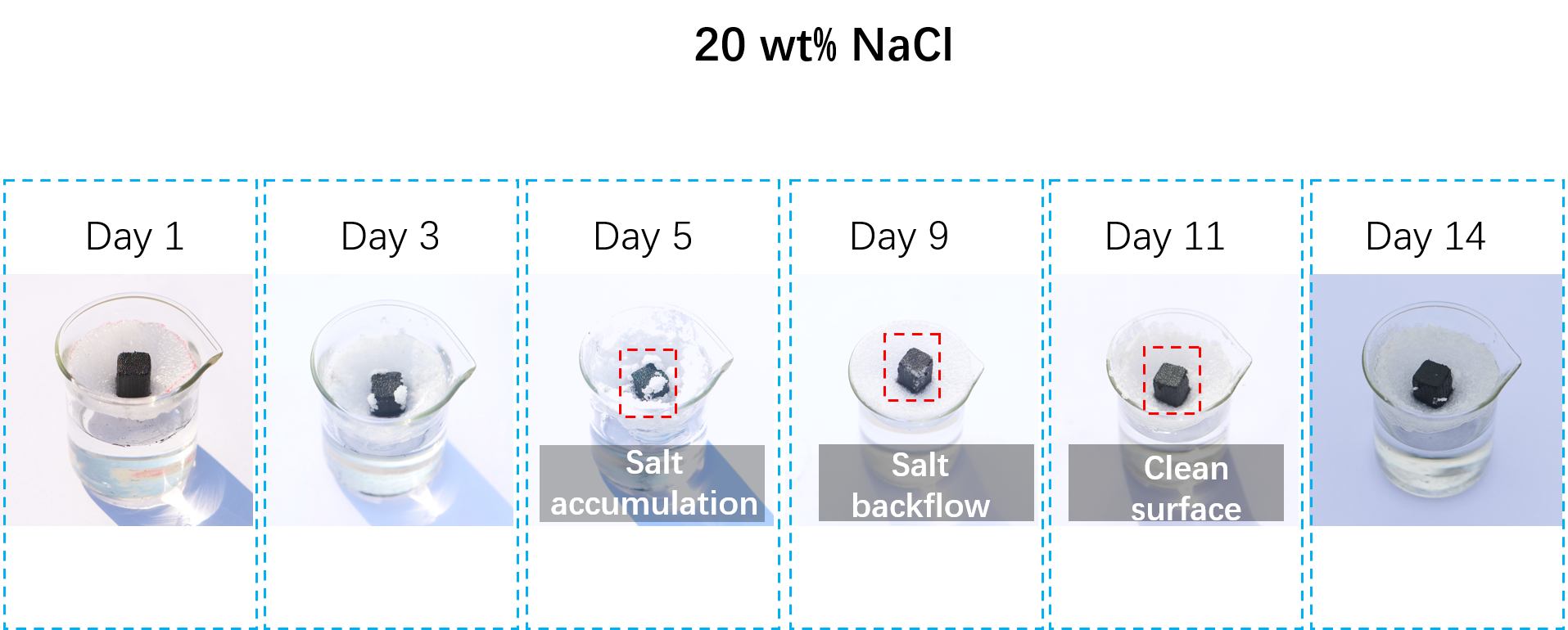
**Figure S16.** 14-day outdoor evaporation in 20 wt% NaCl brine, Chengdu, China; daily photographs at 9:30 a.m.


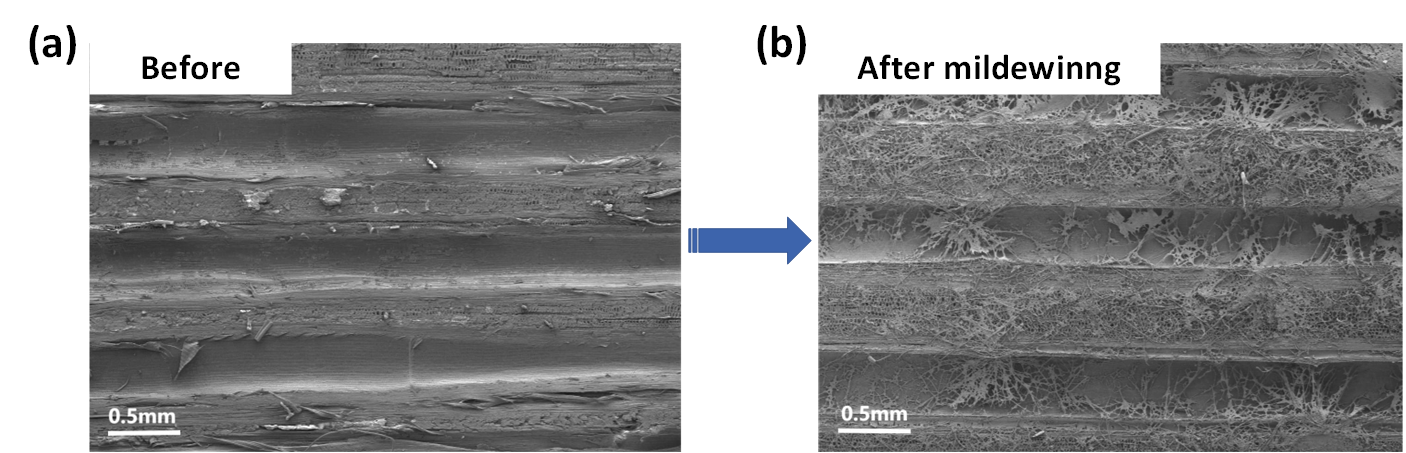


**Figure S17.** Comparative SEM analysis of NR: (a) pristine state; (b) post-mildewing morphology.


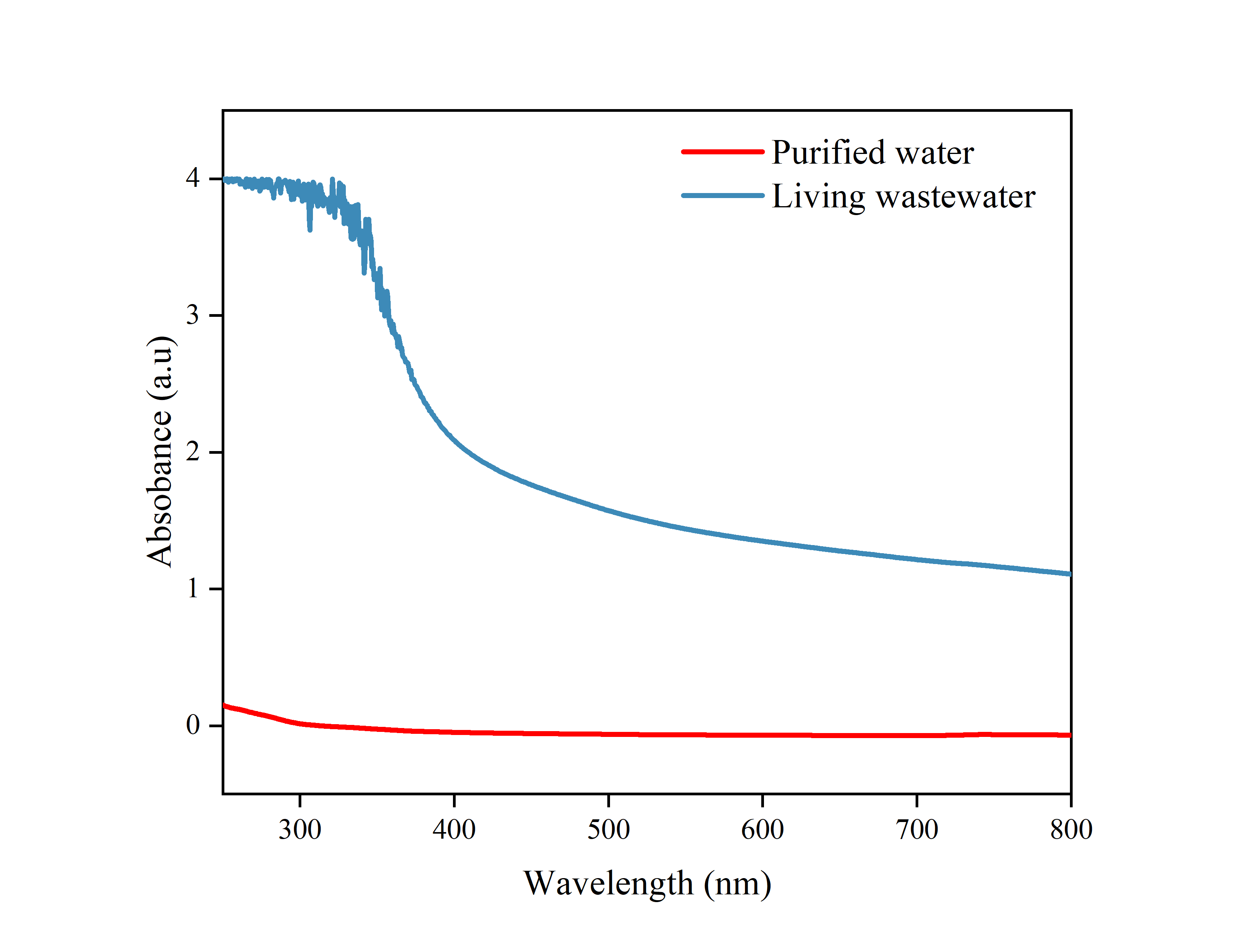


**Figure S18.** UV-vis absorption spectra of real food-waste swill before and after purification.


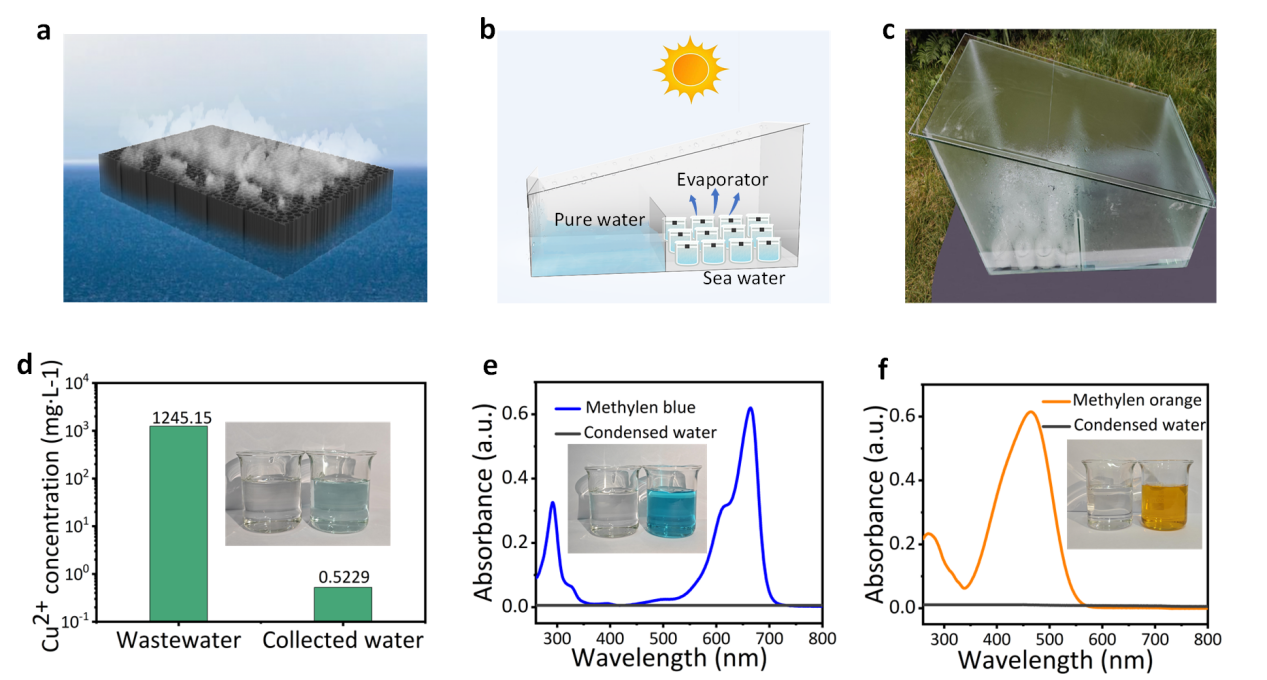


**Figure S19.** a, b) Schematic illustrations of the P-CDR-700 array for practical solar-driven desalination applications. c) Photographic image of the experimental setup corresponding to panel b. d) Cu²⁺ ion removal efficiency from simulated heavy metal-contaminated water using P-CDR-700 purification (inset: visual comparison of untreated and purified solutions). e, f) UV-vis absorption spectra of e) methylene blue and (f) methyl orange solutions before and after purification, with insets showing corresponding solution color evolution.

Solutions containing specific concentrations of copper ions, methyl orange, and methyl blue were prepared to simulate heavy metal wastewater and dye wastewater, respectively. P-CDR-700 was then used to purify these wastewater samples (Figure S19d). As shown in Figure S19d, after solar-driven evaporation, the color of the copper ion wastewater changed from turquoise to nearly colorless, with a removal rate of 99.958 %, demonstrating significant effectiveness. As depicted in Figure S19e and Figure S19f, the UV-visible absorption peaks for methyl blue (around 291 and 665 nm) and methyl orange (around 273 and 465 nm) disappeared, and the treated wastewater became colorless and clear, indicating effective removal of organic pollutants. This excellent water purification capability of P-CDR-700 is mainly attributed to its abundant porous structure, which provides a large specific surface area, increasing contact opportunities with metal ions and enhancing adsorption capacity.


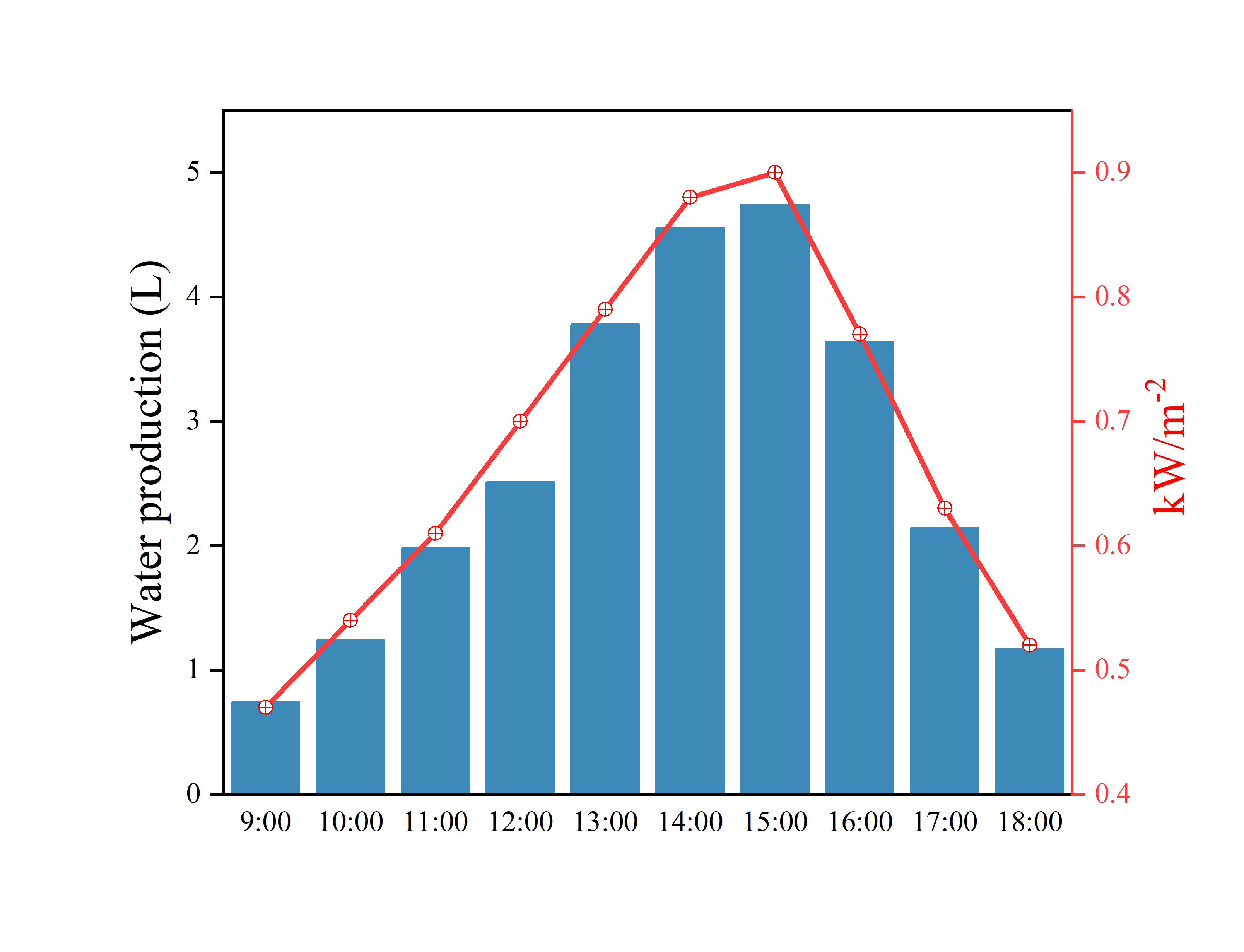


**Figure S20.** Demonstration of modular scalability: a 5×5 array of P-CDR-700 evaporators during outdoor operation, producing ~24.49 L of freshwater from 9:00 to 18:00.

**4. Tables**

Table S1. Comparison with other SIE

| Materials | Evaporation efficiency  (kg·m^-2·^h^-1^) | Salt resistance  (kg·m^-2·^h^-1^) | Mildew  days | Procedure | Ref. |
| --- | --- | --- | --- | --- | --- |
| WAISE  (3D biomass) | 3.3 (1 sun) | _ | _ | 4 steps | [32] |
| Loofah vine aerogel  (Aerogel) | 3.26 (1 sun) | _ | _ | 5 steps | [2] |
| HPCS/DF-PEG  (Hydrogel) | 3.21 (1 sun) | _ | _ | 5 steps | [34] |
| (CD)-based evaporator  (3D biomass) | 2.93 (1 sun) | _ | _ | 4 steps | [30] |
| CDs-Wood  (3D biomass) | 2.86 (1 sun) | _ | _ | 3 steps | [25] |
| CoFe2O4/CNT/bamboo  fabrics  (Membrane) | 2.72 (1 sun) | _ | _ | 4 steps | [19] |
| ZIF-8@PDA@wood  (3D biomass) | 2.7 (1 sun) | _ | _ | 4 steps | [14] |
| CS/BFs/CPPs  (3D biomass) | 2.317 (1 sun) | _ | _ | 3 steps | [18] |
| Carbonized durian peel  (3D biomass) | 2.22 (1 sun) | _ | _ | 4 steps | [5] |
| GO  (Membrane) | 2.05 (1 sun) | _ | _ | 4 steps | [38] |
| Carbonized bamboo  (3D biomass) | 2.034 (1 sun) | _ | _ | 4 steps | [16] |
| lignin/wood based  (3D biomass) | 1.93 (1sun) | _ | _ | 4 steps | [10] |
| Plantain cellulose@HCT  (Aerogel)  (3D biomass) | 1.86 (1 sun) | _ | _ | 6 steps | [9] |
| FCC  (Janus) | 1.66 (1 sun) | _ | _ | 4 steps | [36] |
| NP-Ag  (Membrane) | 1.42 (1 sun) | _ | _ | 4 steps | [37] |
| Au-rGO/wood aerogel  (Aerogel) | 1.39 (1 sun) | _ | _ | 4 steps | [23] |
| Black melanin  nanoparticles@MIL-53(Fe)  (Membrane) | 1.34 (1 sun) | _ | _ | 4 steps | [4] |
| Carbonized pleurotus eryngii  (3D biomass) | 1.22 (1 sun) | _ | _ | 4 steps | [7] |
| TWA  (Aerogel) | 1.17(1 sun) | _ | _ | 3 steps | [22] |
| 2D@3D Janus evaporators  (Janus) | 1.04 (1 sun) | _ | _ | 4 steps | [27] |
| EC-PPy-MO  (Foam) | 1.8 (1 sun) | 2.8  (25 wt% NaCl) | _ | 5 steps | [13] |
| Ni-NCNTs/KF  (3D biomass) | 3.22 (1 sun) | 2.96  (20 wt% NaCl) | _ | 3steps | [31] |
| FAS-MC@M  (Membrane) | 3.2 (1 sun) | 2.53  (20 wt% NaCl) | _ | 5 steps | [1] |
| HEA-BW  (3D biomass) | 2.58 (1 sun) | 1.65  (20 wt% NaCl) | _ | 4 steps | [15] |
| PC-CM  (3D biomass) | 2.06(1 sun) | 0.78  (20 wt% NaCl) | _ | 4 steps | [26] |
| CWG  (3D biomass) | 1.76 (1 sun) | 1.44  (20 wt% NaCl) | _ | 4 steps | [29] |
| WSUSE | 1.53(1 sun) | 1.45  (20 wt% NaCl) | - | 5 steps | [8] |
| CNT@WS evaporator  (3D biomass) | 1.43 (1 sun) | 1.43  (20 wt% NaCl) | _ | 4 steps | [28] |
| PDA@PVA@PTFE (Hydrogel) | 2.01 (1 sun) | 1.91  (15 wt% NaCl) | _ | 4 steps | [33] |
| CDs/PPy-CPP  (3D biomass) | 2.46 (1 sun) | 2.46  (3.5 wt% NaCl) | _ | 2 steps | [6] |
| CTP  (Janus) | 2.12 (1 sun) | 2.06  (3.5 wt% NaCl) | _ | 5 steps | [35] |
| birch alkali lignin based double-layer biomass (Aerogel) | 2.06 (1 sun) | 2 kg  (3.5 wt% NaCl) | _ | 4 steps | [11] |
| PAAS-carbonized loofah  (Hydrogel) | 1.83 (1 sun) | 1.71  in seawater | _ | 4 steps | [3] |
| 1. COFs   (3D biomass) | 1.79 (1 sun) | _ | _ | 4 steps | [12] |
| DW-TA-Fe3+  (3D biomass) | 1.79 (1 sun) | _ | 30 days | 4 steps | [17] |
| AC-BS  (3D biomass) | 1.51(1 sun) | 1.43  (20 wt% NaCl) | 7 days | 7 steps | [24] |
| surface-carbonized rattan  (3D biomass) | 1.47 (1 sun) | 1.17  (20 wt% NaCl) | _ | 2 steps | [20] |
| Carbonized rattan  (3D biomass) | 1.47 (1 sun) | 0.54  (25 wt% NaCl) | _ | 2 steps | [21] |
| **Programmed carbonized rattan**  **(biomass material)** | **3.34 (1 sun)** | **1.76**  **(20wt% NaCl);**  **14-day outdoor desalination of 20% NaCl brine** | **23 days** | **3steps** | **This work** |

**“_” indicates that the property is either not mentioned or not applicable.**

References in this table can be found at the end of this file.

Table S2. COMSOL Input Parameters for P-CDR-700

| Name | Expression | Unit | Value | Description |
| --- | --- | --- | --- | --- |
| T0 | 293.15 | [K] | 293.15 K | Ambient temperature |
| phi_0 | 0.1 |  | 0.1 | Ambient relative humidity |
| phi_1 | 0.985 |  | 0.985 | Relative humidity |
| S_il | 0.1 |  | 0.1 | Irreductible liquid phase saturation |
| por | 0.75 |  | 0.75 | Porosity |
| kappa | por/8*Rc^2 |  | 5.7038E-14 m² | Penetration rate |
| k_s | 0.1462 | [W/(m*K)] | 0.1462 W/(m·K) | Heat transfer coefficient |
| cp_s | 1970 | [J/(kg*K)] | 1970 J/(kg·K) | Specific heat capacity |
| rho_s | 288 | [kg/m^3] | 288 kg/m³ | Density |
| Rc | 0.00000078 | [m] | 7.8E-7 m | Pore radius |

Table S3. COMSOL Input Parameters for Un-CDR-700

| Name | Expression | Unit | Value | Description |
| --- | --- | --- | --- | --- |
| T0 | 293.15 | [K] | 293.15 K | Ambient temperature |
| phi_0 | 0.1 |  | 0.1 | Ambient relative humidity |
| phi_1 | 0.985 |  | 0.985 | Relative humidity |
| S_il | 0.1 |  | 0.1 | Irreductible liquid phase saturation |
| por | 0.88 |  | 0.88 | Porosity |
| kappa | por/8*Rc^2 |  | 6.6924E-14 m² | Penetration rate |
| k_s | 0.1529 | [W/(m*K)] | 0.1529 W/(m·K) | Heat transfer coefficient |
| cp_s | 1650 | [J/(kg*K)] | 1650 J/(kg·K) | Specific heat capacity |
| rho_s | 141.9 | [kg/m^3] | 141.9 kg/m³ | Density |
| Rc | 0.00000078 | [m] | 7.8E-7 m | Pore radius |

**Reference**

[1] Y. Zheng, Y. Lian, H. Bao, H. Guo, Y. Hu, J. Zhao, H. Zhang, *Chem. Eng. J.* **2023**, *472*, 145003.

[2] Y. Lv, R. Xu, K. Zhang, L. Hong, J. Zhou, B. Weng, Z. Huang, S. Wu, Y. Guo, Y. Chen, *J. Cleaner Prod.* **2023**, *402*, 136817.

[3] X. Wang, L. Zhang, D. Zheng, X. Xu, B. Bai, M. Du, *Chem. Eng. J.* **2023**, *462*, 142265.

[4] J. Wang, Z. Zhao, C. Yang, M. Sun, J. Chen, Y. Zhou, H. Xu, *Desalination.* **2023**, *556*, 116577.

[5] L. Zeng, D. Deng, L. Zhu, H. Wang, Z. Zhang, Y. Yao, *Energy.* **2023**, *273*, 127170.

[6] L. Sun, X. Zhang, H. Yuan, H. Cong, Y. Shi, J. Lu, W. Shi, F. Guo, *Chem. Eng. J.* **2023**, *477*, 147279.

[7] Z. Zhou, J. Gong, C. Zhang, W. Tang, B. Wei, J. Wang, Z. Fu, L. Li, W. Li, L. Xia, *Renewable Energy.* **2023**, *216*, 118987.

[8] H. Zhang, X. Li, X. Liu, Y. Du, W. Xie, S. Zheng, L. Yang, J. Shi, D. Jing, *Chem. Eng. J.* **2023**, *473*, 145484.

[9] J. Wu, X. Yang, X. Jia, J. Yang, X. Miao, D. Shao, H. Song, Y. Li, *Chem. Eng. J.* **2023**, *471*, 144684.

[10] Y. Gu, D. Wang, Y. Gao, Y. Yue, W. Yang, C. Mei, X. Xu, Y. Xu, H. Xiao, J. Han, *Adv. Funct. Mater.* **2023**, *33* (43), 2306947.

[11] Q. Shao, Y. Luo, M. Cao, X. Qiu, D. Zheng, *Chem. Eng. J.* **2023**, *476*, 146678.

[12] W. Kong, Y. Ma, H. Huang, X. Sun, T. Ma, *Chem. Eng. J.* **2025**, *520*, 166125.

[13] Y. Kong, Y. Gao, B. Gao, Y. Qi, W. Yin, S. Wang, F. Yin, Z. Dai, Q. Yue, *Chem. Eng. J.* **2022**, *445*, 136701.

[14] Y. Lu, D. Fan, Z. Shen, H. Zhang, H. Xu, X. Yang, *Nano Energy.* **2022**, *95*, 107016.

[15] Y. Li, Y. Ma, Y. Liao, L. Ji, R. Zhao, D. Zhu, X. Hu, G. Qin, H. Rong, X. Zhang, *Adv. Energy Mater.* **2022**, *12* (47), 2203057.

[16] P. Zhang, C. Sheng, M. Xie, Z. Wang, C. Jin, *Ind. Eng. Chem. Res.* **2023**, *62* (13), 5574.

[17] M. Xie, P. Zhang, Y. Cao, Y. Yan, Z. Wang, C. Jin, *npj Clean Water.* **2023**, *6* (1), 12.

[18] X. Sun, X. Jia, J. Yang, S. Wang, Y. Li, D. Shao, H. Song, *J. Mater. Chem. A.* **2021**, *9* (42), 23891.

[19] R. J. Pan, J. Wu, J. Qu, T. Zhang, F. Z. Jiao, M. Zhao, M. Y. Han, X. Li, Z. Z. Yu,  *J. Mater. Sci. Technol.* **2024**, *179*, 40.

[20] R. Yang, F. Liu, L. Xia, X. Wu, X. Zhang, F. Guo, Y. Yu, *Sep. Purif. Technol.* **2022**, *286*, 120412.

[21] C. Dang, X. Zhang, L. Huang, G. Xu, L. Gu, X. Cao, M. Zhu, *Desalination.* **2023**, *550*, 116408.

[22] T. Meng, B. Jiang, Z. Li, X. Xu, D. Li, J. Henzie, A. K. Nanjundan, Y. Yamauchi, Y. Bando, *Nano Energy.* **2021**, *87*, 106146.

[23] Q. Zhang, L. Li, B. Jiang, H. Zhang, N. He, S. Yang, D. Tang, Y. Song, *ACS Appl. Mater. Interfaces.* **2020**, *12* (25), 28179.

[24] Y. Chen, J. Fang, T. Ling, M. Xia, P. Xu, Y. Cao, D. Wei, J. Gao, *Desalination.* **2022**, *541*, 116003.

[25] H. Li, W. Zhu, M. Li, Y. Li, R. T. K. Kwok, J. W. Y. Lam, L. Wang, D. Wang, B. Z. Tang, *Adv. Mater.* **2021**, *33* (36), 2102258.

[26] H. Xu, Y. She, F. Tian, N. Su, X. Xu, *Chem. Eng. J.* **2024**, *481*, 148680.

[27] Y. Kuang, C. Chen, S. He, E. M. Hitz, Y. Wang, W. Gan, R. Mi, L. Hu, *Adv. Mater.* **2019**, *31* (23), 1900498.

[28] X. Dai, H. Guan, X. Wang, M. Wu, J. Hu, X. Wang, *ACS Appl. Mater. Interfaces.* **2023**, *15* (31), 38100.

[29] W. Wang, Z. Tian, N. He, X. Huan, J. Fan, Y. Li, *Desalination.* **2024**, *574*, 117232.

[30] W. Ma, W. Cao, M. Cui, H. Lu, R. Xiong, C. Huang, *Chem. Eng. J.* **2023**, *478*, 147404.

[31] L. Ying, Z. Yuan, J. Ding, W. Wang, S. Liu, J. Lu, *Chem. Eng. J.* **2024**, *487*, 150593.

[32] J. Wu, Z. Cui, Y. Yu, B. Yue, J. Hu, J. Qu, J. Li, D. Tian, Y. Cai, *Adv. Sci.* **2023**, *10* (35), 2305523.

[33] N. Li, X. Zhang, J. Zhu, S. Xiong, Y. Xu, Y. Li, *Nano Lett.* **2025**, *25* (25), 10072.

[34] Y. Liu, Y. Tian, N. Liu, S. Zhao, H. Zhai, J. Ji, W. Cao, L. Tao, Y. Wei, L. Feng, *Small.* **2024**, *20* (4), 2305903.

[35] X. Dai, H. Guan, X. Wang, M. Wu, P. Jiang, Y. Chai, X. Wang, *Chem. Eng. J.* **2024**, *499*, 155796.

[36] Z. Yang, C. Kang, Y. Lu, Y. Tian, Y. Lyu, T. Yu, M. Yi, X. Wang, Z. Zhao, Y. J. Zeng, J. Lu, *Solar Energy.* **2023**, *265*, 112151.

[37] B. Yu, Y. Wang, Y. Zhang, Z. Zhang, *Nano Research.* **2023**, *16* (4), 5610.

[38] T. Yang, H. Lin, K. T. Lin, D. Mesa Saldarriaga, G. Yang, C. Guo, H. Zhang, J. Zhang, S. Fraser, A. K. T. Lau, T. Ma, B. Jia, *Carbon.* **2022**, *199*, 469.
